# Supplementary material for: Development of Accurate Long-lead COVID-19 Forecast
Source: PLoS Comput Biol. 2023 Jul 17;19(7):e1011278. doi: 10.1371/journal.pcbi.1011278 (PMC10374152; doi:10.1371/journal.pcbi.1011278)
Supplement: S1 Text — (DOCX) [file pcbi.1011278.s001.docx]

**S1 Text**

**Supplemental Information**

**for**

**Development of Accurate Long-lead COVID-19 Forecast**

Wan Yang and Jeffrey Shaman

This document includes:

Supplemental Methods

Supplemental Tables A – H

**SUPPLEMENTAL METHODS**

**Data sources and processing**

For model calibration, we used reported COVID-19 case and mortality data to capture transmission dynamics, mobility data to represent concurrent NPIs, and vaccination data to account for changes in population susceptibility due to vaccination. State level COVID-19 case and mortality data were sourced from the New York Times (NYT) (1) and included all variants. In our previous studies, overall case/mortality data were sufficient to estimate key epidemiological parameters when different VOC waves were separated in time (2-4); however, in the US, the Omicron BA.1 wave overlapped substantially with the Delta wave during November 2021 – January 2022, making inference challenging. Thus, here we separated the forecasts into two periods (i.e., a pre-Omicron period combining all non-Omicron variants, and an Omicron period combining all Omicron subvariants) and used variant-specific case and mortality data for model training and forecast evaluation for each period. Specifically, we used variant proportion data sourced from GISAID (5) and compiled by CoVariants.org (6) to compute the weekly number of cases and deaths due to non-Omicron variants and Omicron, separately (for simplicity, loosely referred to as variant-specific case and mortality data). Because only biweekly variant proportion data at the state level were available from CoVariants.org, we used a spline function to impute weekly variant proportion. To compute weekly variant-specific cases, we multiplied the NYT weekly case data by the estimated weekly variant proportion for the same week. To compute weekly variant-specific deaths, we multiplied the NYT weekly mortality data by the estimated weekly variant proportion three weeks later (i.e., assuming a 3-week lag from case detection to death; note the 3-week lag was based on the approximate time lag between the peaks of incidence and mortality time series).

Mobility data were derived from Google Community Mobility Reports (7); we aggregated all business-related categories (i.e., retail and recreational, grocery and pharmacy, transit stations, and workplaces) in all locations in each state to weekly intervals. State level COVID-19 vaccination data were sourced from Our World in Data (8, 9). For models including seasonality, weather data (i.e., temperature and humidity) were used to estimate infection seasonality trends. Hourly surface station temperature and relative humidity came from the Integrated Surface Dataset (ISD) maintained by the National Oceanic and Atmospheric Administration (NOAA) and are accessible using the “stationaRy” R package (10, 11). We computed specific humidity using temperature and relative humidity per the Clausius-Clapeyron equation (12). We then aggregated these data for all weather stations in each state with measurements since 2000 and calculated the average for each week of the year during 2000-2020.

**Modeling variant-specific vaccine effectiveness (VE) and waning vaccine protection against infection**

As noted in the main text, the epidemic model in Eqn 1 includes vaccination and waning vaccine protection. Specifically, vaccination including boosters is represented using the term $\sum_{k=1}^{k=K} v_{k,t}$, where $\upsilon_{k,t}$ is the number of individuals immunized at time *t*, after the *k*-th dose (*k* = 1,…,3 for 2 primary and 1 booster dose here, excluding those immunized after previous doses). We computed $\upsilon_{k,t}$ using vaccination data and adjusted for the delay in antibody development (here, 14 days for the 1^st^ dose and 7 days for subsequent doses) and variant specific VE (13-17). Note that while the 2^nd^ booster dose has been administered for a subset of the population, such data have not been made publicly available and thus not included in our model. Further, given the 2^nd^ dose and 3^rd^ dose (i.e. 1^st^ booster) were administered ~6 months apart (i.e., beyond the estimated VE duration against infection (16)), here we combined data for these two doses. In doing so, we have simplified the model and implicitly assumed that the 3^rd^ dose resumed VE against infection to a level similar to the 2^nd^ dose, as the same VE was applied (Table H).

The model further accounts for waning of vaccine protection against infection, using the term $\sum_{\tau=0}^{\tau=T} {\rho_{\tau}V}_{t-\tau}$. We computed the total number who were vaccinated *τ* days ago and lost protection on day-*t* (*V_t-τ_*) per the VE waning probability (*ρ_τ_*). The probabilities *ρ_τ_* for time *τ* =0, …, T (T= the maximum duration from the earliest vaccination rollout; Table H) were calculated using VE duration data (16) and *V_t-τ_* was computed per line 5 of Eqn 1.

**Observation model to account for under-detection and time-lags in COVID-19 outcomes**

We computed the number of cases and deaths each week using the model-simulated number of infections occurring each day to match with the observations, as done in Yang et al. (18). Briefly, we included 1) a time-lag from infectiousness to detection (i.e., an infection being diagnosed as a case), drawn from a gamma distribution with a mean of *T_d,mean_* days and a standard deviation of *T_d, sd_* days, to account for delays in detection; 2) an infection-detection rate (*r_t_*), i.e. the fraction of infections (including subclinical or asymptomatic infections) reported as cases, to account for under-detection; 3) a time-lag from infectiousness to death; and 4) an infection-fatality risk (*IFR_t_*). Each week, the infection-detection rate (*r_t_*), infection-fatality risk (*IFR_t_*), and the two time-to-detection parameters (*T_d, mean_* and *T_d, sd_*) were estimated along with other parameters (see main text). The time-lag from infectiousness to death during the pre-Omicron period was drawn from a gamma distribution with a mean of 14 days and a standard deviation of 14 days, roughly based on data from New York City (unpublished work). For the Omicron period, many deaths were identified posthumously; thus, it is difficult to estimate the time-lag from infection to death for Omicron infections. Here, based on the slightly longer time-lag between the peaks of case and mortality time series during the Omicron period, we assumed a gamma distribution with a mean of 24 days (i.e., assuming an additional 10-day lag) and a standard deviation of 14 days.

To compute the model-simulated number of new cases each week, we multiplied the model-simulated number of new infections per day by the infection-detection rate, and further distributed these simulated cases in time per the distribution of time-from-infectiousness-to-detection. Similarly, to compute the model-simulated deaths per week and account for delays in time to death, we multiplied the simulated-infections by the IFR and then distributed these simulated deaths in time per the distribution of time-from-infectious-to-death. We then aggregated these daily numbers to weekly totals to match with the weekly case and mortality data for model inference, as described in the main text.

**Settings for anticipating the impact of new variants (the new variant approach)**

The uncertainty due to the possible emergence or surge of new variants in the future is a major challenge for long-lead COVID-19 forecast. To address this challenge, we devised a set of heuristics to anticipate the likely timing and impact of new variant emergence during the forecast period (i.e., the new variant approach). For the very near future (1- to 5 weeks), we used available genomic sequencing data (see “Data sources and processing”). Specifically, we first estimated the growth rate for each circulating variant based on variant proportion 6- to 2 weeks prior to the week of forecast initiation (i.e., assuming a 2-week lag for genomic data collection); for simplicity, we used a log-linear model [i.e., log(variant proportion during week-*t*) ~ week-*t*]. If any variant had a high growth rate (here, arbitrarily set to 10% per week), we deemed it a rising variant that could further affect the population susceptibility and overall virus transmissibility. To anticipate its impact, we then used a smoothing spline to 1) project when the variant would reach a 100% proportion and 2) project the number of weeks for the variant to grow from 0% to 100%. The first estimate was then used to set the timing of the continued impact and the second estimate was used to scale the increases in population susceptibility. Here, arbitrarily, we assumed a baseline of 1.5 – 4.5% increase in susceptibility for each week the new variant increased in proportion; however, if the estimated growth rate (*g*) was >20%, to account for the faster growth, we scaled that baseline by a factor of (1+*g*)/1.2. While the growth advantage of a new variant could also come from increased transmissibility, for simplicity, here we opted to solely adjust for population susceptibility. In addition, given the fast displacement of new variants, we opted not to use projected estimates 5 weeks beyond the forecast week, i.e. these changes were only applied to the first 5 weeks of a forecast.

When genomic data could not be used (i.e. beyond the first 5 forecast weeks or when genomic data were not available), we used the following heuristics to anticipate the likely timing and impact of a new variant surge:

i) New variants tend to emerge after a recent large wave (here, defined arbitrarily as a 25% attack rate over 3 months for the non-Omicron period, and a 33% attack rate over 2 months for the Omicron period). We identified these times using estimated/forecasted infection rates during the preceding months and the forecast period.

ii) New variants tend to emerge and/or become widespread during northern hemisphere winter (December – February), southern hemisphere winter (June – August), and/or the monsoon season (e.g. June – September in India) and could be introduced to the US during these months. We identified these times using calendar month.

iii) During the above times with potential new variant emergence, the population susceptibility and virus transmissibility could increase. Accordingly, to account for susceptibility changes, we resampled half of the model ensemble to increase the population susceptibility by 2-9% for weeks flagged per the conditions described in i and ii. However, this susceptibility increase was only triggered when the mean population susceptibility was below 40% to avoid over-adjustment. Similarly, to avoid the system being trapped in an outbreak-begets-outbreak cycle, no further adjustments were made to susceptibility if a wave had been forecast the prior weeks or the cumulative adjustment had exceeded a threshold (here, set to 40% of the population over 26 weeks for pre-Omicron period and 60% for the Omicron period). To account for transmissibility changes, we expanded the variance of the transmission rate (i.e., $\beta_{t}$ in Eqn 1) by applying an inflation factor of 1.3 (pre-Omicron period) or 1.1 (Omicron period) to ensemble members falling between the 50^th^ and 95^th^/90^th^ (pre-Omicron/Omicron period) percentiles (i.e., the ones with higher but not too extreme values) for weeks identified per the conditions described in i and ii.

**The fixed seasonality model**

The fixed seasonality model represents the dependency of respiratory virus survival, including that of SARS-CoV-2, to temperature and humidity (19, 20) per the following equations:

$R_{0}\left( t \right)=[a_{0}q^{2}\left( t \right)+a_{1}q\left( t \right)+a_{2}]{[\frac{T_{c}}{T\left( t \right)}]}^{T_{exp}}$ (Eqn 3a)

$b_{t}=\frac{R_{0}(t)}{\bar{R_{0}(t)}}$ (Eqn 3b)

As described previously (3, 4), the seasonality function in Eqn 3a assumes that humidity has a bimodal effect on seasonal risk of infection, with both low and high humidity conditions favoring transmission [i.e., the parabola in the 1st set of brackets, where *q*(*t*) is weekly specific humidity measured by local weather stations and *t* = 1,…,52, i.e., week 1 to week 52 of the year]; this effect is further modulated by temperature, with low temperatures promoting transmission and temperatures above a certain threshold limiting transmission [i.e., the 2nd set of brackets, where *T*(*t*) is weekly temperature measured by local weather stations and *T_c_* is the threshold]. As SARS-CoV-2 specific parameters ($a_{0}$, $a_{1}$, $a_{2}$, *T_c_*, and *T_exp_* in Eqn 3a) are not available, we used parameters estimated for influenza (21) and scaled the weekly outputs [i.e., $R_{0}\left( t \right)$] by the annual mean (i.e., $\bar{R_{0}}$) per Eqn 3b, as done in Yang and Shaman (4). In doing so, the scaled outputs (*b_t_*) are no longer specific to influenza; rather, they represent the *relative*, seasonality-related transmissibility by week, general to viruses sharing similar seasonal responses. The estimated relative seasonal trend, *b_t_*, is then used to adjust the relative transmission rate at time *t* in Eqn 1.

**The transformed seasonality model**

The transformed seasonality model transforms the *b_t_* estimates from Eqn 3b to allow flexibility in the seasonal trend. To do so, we include three parameters to fine tune the peak of the seasonal trend ($p_{shift}$; i.e., the number of weeks earlier or later than the peak estimated for influenza), the number of weeks during a year with *b_t_* >1 ($\delta$; i.e., the duration with elevated infection risk), and another parameter $b_{t, lwr}$ that adjusts the lowest *b_t_* value. Specifically, the transformation first adjusts values of *b_t_* greater than 1, by shifting the timing by $p_{shift}$ weeks and adjusting the duration with elevated infection risk to $\delta$, per

$b_{t\left\{ b_{t}>1 \right\}}^{'}=\frac{b_{t\left\{ b_{t}>1 \right\}+p_{shift}}}{\frac{n_{b_{t}>1}}{\delta}}$ (Eqn 4a)

where $n_{b_{t}>1}$ is the number of weeks with *b_t_* >1 during the 1-year cycle. For weeks with *b_t_* ≤1, the transformation adjusts the values, by shifting the timing by $p_{shift}$ weeks and adjusting the duration with lower infection risk to $52-\delta$, per

$b_{t\left\{ b_{t}\leq1 \right\}}^{'}=\frac{b_{t\left\{ b_{t}\leq1 \right\}+p_{shift}}}{n_{b_{t}\leq1}/\left( 52-\delta\right)}$ (Eqn 4b)

The approach then further scales $b_{t\left\{ b_{t}\leq1 \right\}}^{'}$ to increase the relative infection risk, per

$b_{t\left\{ b_{t}\leq1 \right\}}^{''}=1-(1-b_{t\left\{ b_{t}\leq1 \right\}}^{'})(min\left\{ 1,\frac{\min\left\{ b_{t\left\{ b_{t}\leq1 \right\}}^{'} \right\}}{b_{t, lwr}} \right\})$ (Eqn 4c)

$b_{t\left\{ b_{t}>1 \right\}}^{''}\equiv b_{t\left\{ b_{t}>1 \right\}}^{'}$ and $b_{t\left\{ b_{t}\leq1 \right\}}^{''}$ are then pooled together and scaled to have a mean of 1 over the 1-year cycle, per

$b_{t}^{'''}=\frac{b_{t}^{''}}{\bar{b_{t}^{''}}}$ (Eqn 4d)

There could be multiple combinations of the three parameters (i.e., $p_{shift}$, $\delta$, and $b_{t, lwr}$). Due to the lack of SARS-CoV-2 data to inform the parameter estimates, here we opted to optimize the range for each parameter (as opposed to estimate specific best-fit parameters). Briefly, we tested 2 levels (low vs. high) for each parameter and thus 8 in combination for each state (S4 Fig). We then identified the best range for each state based on forecast performance during the 2^nd^ wave, i.e., before the surge of SARS-CoV-2 VOCs to minimize potential confounding. The best parameter ranges (S4 Fig) were then used in the transformed seasonality model in the main analysis.

**Additional details on the retrospective forecast and forecast evaluation**

As noted in the main text, retrospective forecasts for the non-Omicron period were done through the week of August 15, 2021. We stopped initiating the non-Omicron forecasts in mid-August 2021 to allow at least a few weeks of Delta-related data to calibrate the model before forecasting the Delta wave. However, a 6-month forecast initiated during mid-June – mid-August 2021 would extend to mid-December 2021 – mid-Feb 2022, when Omicron BA.1 had become predominant, depending on location; this overlap would lead to lower forecast accuracy, since here we did not account for the emergence of Omicron BA.1 and fast displacement of Delta. Given the low number of Delta-associated cases/deaths in 2022, weekly targets (i.e., 1- to 26- week ahead prediction) for weeks in 2022 were excluded from the evaluation; however, as Delta was the main circulating variant during the 6-month period for these forecasts, all the overall targets (i.e., peak week, peak intensity, and cumulative total) were evaluated based on Delta-specific data and included in the analysis.

Both the model inference and forecast were run with *n* = 500 model realizations (i.e., ensemble members). The ensemble and its distribution provided probabilistic forecasts for 4 types of targets here, i.e., 1-to 26-week ahead prediction, peak intensity, peak week, and cumulative totals over the entire 26-week forecast period. For example, for the 1-week ahead prediction ($c_{t+1}$), the fraction of ensemble members falling in a given bin $[c_{i},c_{i+1})$ can be used to represent the forecast probability density, i.e., $\Pr\left( c_{t+1}\in[c_{i},c_{i+1}) \right)=n_{\left\{ c_{t+1}\geq c_{i} \&c_{t+1}<c_{i+1} \right\}}/n$. Similarly, for the peak week prediction, predicted peak week by individual ensemble members (*p_w_* = 1, 2, …, 26) can be aggregated and the distribution can be used to represent the probability distribution of the forecast, i.e., $\Pr\left( p_{w}=w \right)=n_{\left\{ p_{w}=w \right\}}/n$.

The forecast probabilities can then be used to compute the log score for evaluation. To do so, we first binned the forecast ensemble to generate the forecast probability distribution $\Pr\left( x \right)$, e.g., $\Pr\left( c_{t+1} \right)$ for 1-week ahead prediction and $\Pr\left( p_{w} \right)$ for peak week. Here, for cases, bins of the weekly targets were set to $\left[ 0, 0.05\% \right)$, $\left[ 0.05\%, 0.1\% \right)$, …, $\left[ 0.95, 1\% \right)$, and $\left[ 1\%, 100\% \right]$ (i.e., increments of 0.05%, or 500 per million people, up to 1% of the population; and the rest combined in the last bin); bins of cumulative cases over 26 weeks were set to $\left[ 0, 2\% \right)$, $\left[ 2\%, 4\% \right)$, …, $\left[ 8\%, 10\% \right)$, $\left[ 10\%, 15\% \right)$, $\left[ 15\%, 20\% \right)$,…,$\left[ 45\%, 50\% \right)$, and $\left[ 50\%, 100\% \right]$ (i.e., increments of 2% up to 10%, then increments of 5% up to 50% of the population; and the rest combined in the last bin). For mortality, bins of the weekly targets were set to $\left[ 0, 0.001\% \right)$, $\left[ 0.001, 0.002 \right)$, …, $\left[ 0.019\%, 0.02\% \right)$, and $\left[ 0.02\%, 100\% \right]$ (i.e., increments of 0.001%, or 10 per million people, up to 0.02% of the population; and the rest combined in the last bin); bins of cumulative deaths over 26 weeks were set to $\left[ 0, 0.02\% \right)$, $\left[ 0.02\%,0.04\% \right)$, …,$\left[ 0.08\%, 0.1\% \right)$, $\left[ 0.1\%, 0.15\% \right)$, $\left[ 0.15\%,0.2\% \right)$,…, $\left[ 0.45\%, 0.5\% \right)$, and $\left[ 0.5\%, 100\% \right]$ (i.e., increments of 0.02% up to 0.1%, then increments of 0.05% up to 0.5% of the population; and the rest combined in the last bin). For the peak week of both cases and deaths, the bin size was set to 1 week. The log score was then computed as:

$\log score=\log[{\Pr\left( x \right)}_{x\in{bin}^{*}}+{\Pr\left( x \right)}_{x\in{bin}^{*-1}}+{\Pr\left( x \right)}_{x\in{bin}^{*+1}}]$ (Eqn 5)

where $\Pr\left( x \right)$ is the forecast probability for target $x$; ${bin}^{*}$ is the bin that contains the observed value for that target (see bin specifications above) and ${bin}^{*-1}$ and ${bin}^{*+1}$ are the two adjacent bins. Note that, here we used smaller bins and deemed ensemble members falling within the bin covering the observation and its two adjacent bins accurate, which is equivalent to using a single larger bin spanning all those smaller bins. However, as the probabilistic forecasts (i.e., probabilities in each bin) were generated and stored before the final evaluation, using smaller bins allowed more flexible post processing and evaluation if needed (e.g., the log score can be computed based on a single small bin if preferred).

**References:**

1. The New York Times (2022) Coronavirus (Covid-19) Data in the United States. <https://github.com/nytimes/covid-19-data>

2. Yang W & Shaman JL (2022) COVID-19 pandemic dynamics in South Africa and epidemiological characteristics of three variants of concern (Beta, Delta, and Omicron). *Elife* 11.

3. Yang W & Shaman J (2022) COVID-19 pandemic dynamics in India, the SARS-CoV-2 Delta variant and implications for vaccination. *J R Soc Interface* 19(191):20210900.

4. Yang W & Shaman J (2021) Development of a model-inference system for estimating epidemiological characteristics of SARS-CoV-2 variants of concern. *Nature Communications* 12:5573.

5. Global Initiative on Sharing All Influenza Data (GISAID) (2021) Tracking of Variants. <https://www.gisaid.org/hcov19-variants/>

6. CoVariants. <https://covariants.org>

7. Google Inc. (2020) Community Mobility Reports. <https://www.google.com/covid19/mobility/>

8. Our World in Data (2022) Data on COVID-19 (coronavirus) vaccinations by Our World in Data. <https://github.com/owid/covid-19-data/blob/master/public/data/vaccinations/us_state_vaccinations.csv>

9. Mathieu E*, et al.* (2021) A global database of COVID-19 vaccinations. *Nature human behaviour* 5(7):947-953.

10. Iannone R (2020) Package ‘stationaRy’. <https://cran.r-project.org/web/packages/stationaRy/stationaRy.pdf>

11. Iannone R (2020) stationaRy. <https://github.com/rich-iannone/stationaRy>

12. Wallace J & Hobbs P (2006) *Atmospheric Science: An Introductory survey* (Academic Press, New York) 2nd Edition Ed p 504.

13. Polack FP*, et al.* (2020) Safety and Efficacy of the BNT162b2 mRNA Covid-19 Vaccine. *New Engl J Med*.

14. Baden LR*, et al.* (2021) Efficacy and Safety of the mRNA-1273 SARS-CoV-2 Vaccine. *N Engl J Med* 384(5):403-416.

15. Haas EJ*, et al.* (2021) Impact and effectiveness of mRNA BNT162b2 vaccine against SARS-CoV-2 infections and COVID-19 cases, hospitalisations, and deaths following a nationwide vaccination campaign in Israel: an observational study using national surveillance data. *The Lancet* 397(10287):1819-1829.

16. UK Heath Security Agency (2022) COVID-19 vaccine surveillance report (Week 17, 28 April 2022). <https://assets.publishing.service.gov.uk/government/uploads/system/uploads/attachment_data/file/1072064/Vaccine-surveillance-report-week-17.pdf>

17. Kirsebom FCM*, et al.* (COVID-19 vaccine effectiveness against the omicron (BA.2) variant in England. *The Lancet Infectious Diseases*.

18. Yang W*, et al.* (2021) Estimating the infection-fatality risk of SARS-CoV-2 in New York City during the spring 2020 pandemic wave: a model-based analysis. *The Lancet. Infectious diseases* 21(2):203-212.

19. Biryukov J*, et al.* (2020) Increasing Temperature and Relative Humidity Accelerates Inactivation of SARS-CoV-2 on Surfaces. *mSphere* 5(4):e00441-00420.

20. Morris DH*, et al.* (2021) Mechanistic theory predicts the effects of temperature and humidity on inactivation of SARS-CoV-2 and other enveloped viruses. *Elife* 10.

21. Yuan H, Kramer SC, Lau EHY, Cowling BJ, & Yang W (2021) Modeling influenza seasonality in the tropics and subtropics. *PLoS Comput Biol* 17(6):e1009050.

**Table A.** Impact of deflation. Numbers show the relative difference in mean log score computed using Eqn 6, or relative difference in mean point prediction accuracy computed using Eqn 7. For each pairwise comparison (e.g., 0.95 vs none), a positive difference indicates the former approach (e.g., 0.95) outperforms the latter (e.g., none).

| Metric | Measure | New variant setting | Seasonality setting | Pairwise comparison of deflation settings | | |
| --- | --- | --- | --- | --- | --- | --- |
|  |  |  |  | 0.95 vs none | 0.9 vs none | 0.9 vs 0.95 |
| Log score | Cases | No new variants | No seasonality | 20.1% | 43.3% | 19.4% |
| Log score | Cases | No new variants | Fixed seasonality | 18.1% | 34.2% | 13.6% |
| Log score | Cases | No new variants | Transformed seasonality | 19% | 36.8% | 15% |
| Log score | Cases | New variants | No seasonality | 20.2% | 38.7% | 15.4% |
| Log score | Cases | New variants | Fixed seasonality | 19.7% | 35% | 12.8% |
| Log score | Cases | New variants | Transformed seasonality | 20% | 35.9% | 13.3% |
| Log score | Deaths | No new variants | No seasonality | 7.67% | 17.2% | 8.85% |
| Log score | Deaths | No new variants | Fixed seasonality | 7.8% | 13.3% | 5.1% |
| Log score | Deaths | No new variants | Transformed seasonality | 7.94% | 14.2% | 5.75% |
| Log score | Deaths | New variants | No seasonality | 7.43% | 15.1% | 7.19% |
| Log score | Deaths | New variants | Fixed seasonality | 8.23% | 13% | 4.45% |
| Log score | Deaths | New variants | Transformed seasonality | 8.4% | 13.2% | 4.45% |
| Accuracy | Cases | No new variants | No seasonality | 28% | 46.5% | 14.4% |
| Accuracy | Cases | No new variants | Fixed seasonality | 30.5% | 38.6% | 6.23% |
| Accuracy | Cases | No new variants | Transformed seasonality | 47.8% | 63.2% | 10.4% |
| Accuracy | Cases | New variants | No seasonality | 20.5% | 32.5% | 10% |
| Accuracy | Cases | New variants | Fixed seasonality | 38.3% | 47.2% | 6.45% |
| Accuracy | Cases | New variants | Transformed seasonality | 47% | 55% | 5.44% |
| Accuracy | Deaths | No new variants | No seasonality | 15.5% | 27% | 9.98% |
| Accuracy | Deaths | No new variants | Fixed seasonality | 20% | 24.6% | 3.84% |
| Accuracy | Deaths | No new variants | Transformed seasonality | 26.8% | 36% | 7.22% |
| Accuracy | Deaths | New variants | No seasonality | 14.7% | 24.1% | 8.18% |
| Accuracy | Deaths | New variants | Fixed seasonality | 26.1% | 34.1% | 6.36% |
| Accuracy | Deaths | New variants | Transformed seasonality | 31.2% | 39.9% | 6.62% |

**Table B.** Impact of new variants settings. Numbers show the relative difference in mean log score computed using Eqn 6, or relative difference in mean point prediction accuracy computed using Eqn 7, by variant wave. A positive number indicates superior performance of the forecast approach with anticipation of new variant emergence.

| Metric | Measure | Seasonality setting | 2nd wave | Alpha | Delta | Omicron |
| --- | --- | --- | --- | --- | --- | --- |
| Log score | Cases | No seasonality | -2.09% | 0.0271% | 66.9% | 10.5% |
| Log score | Cases | Fixed seasonality | -0.573% | -0.33% | 119% | 36.6% |
| Log score | Cases | Transformed seasonality | -1.07% | -1.03% | 93.7% | 34.1% |
| Log score | Deaths | No seasonality | -1.7% | 0.00306% | 19.1% | -5.31% |
| Log score | Deaths | Fixed seasonality | -0.387% | -0.291% | 34.1% | -2.27% |
| Log score | Deaths | Transformed seasonality | -0.918% | -0.651% | 27.8% | -2.73% |
| Accuracy | Cases | No seasonality | -1.52% | -0.11% | 89.1% | 37.1% |
| Accuracy | Cases | Fixed seasonality | 0.578% | 2.16% | 16.8% | 95.8% |
| Accuracy | Cases | Transformed seasonality | -1.33% | 1.96% | 26.1% | 77.8% |
| Accuracy | Deaths | No seasonality | -1.45% | 0.121% | 69.9% | 40.2% |
| Accuracy | Deaths | Fixed seasonality | -0.278% | 2.73% | 15.9% | 67.7% |
| Accuracy | Deaths | Transformed seasonality | -1.31% | 2.47% | 22.5% | 63.2% |

**Table C.** Impact of seasonality, aggregated over all 10 states. Numbers show the relative difference in mean log score or point prediction accuracy, the median of pair-wise difference in log score (95% CI of the median); asterisk (*) indicates if the median is significantly >0 or <0 at the α = 0.05 level, per a Wilcoxon rank sum test. A positive difference indicates superior log score or point prediction accuracy of the first listed approach; a negative difference indicates superior log score or point prediction accuracy of the second listed approach.

| Wave | Season | Metric | Measure | Fixed vs no seasonality | Transformed vs no seasonality | Transformed vs fixed seasonality |
| --- | --- | --- | --- | --- | --- | --- |
| All | All | Log score | Cases | -4.45%, -0.0075 (-0.012, -0.0034)* | -2.8%, -0.006 (-0.01, -0.002)* | 1.73%, 0.01 (0, 0.01)* |
| All | All | Log score | Deaths | 12.7%, 0.04 (0.04, 0.04)* | 14.2%, 0.04 (0.04, 0.04)* | 1.32%, -0.0015 (-0.002, -0.00054)* |
| All | All | Accuracy | Cases | 20.4%, 10% (10%, 10%) | 26.3%, 10% (10%, 10%) | 4.92%, 0% (0%, 5%)* |
| All | All | Accuracy | Deaths | 15.8%, 10% (9.99%, 10%) | 18.2%, 10% (10%, 10%) | 2.09%, 0% (0%, 0%)* |
| All | Respiratory season | Log score | Cases | 39.8%, 0.19 (0.18, 0.2) | 42.3%, 0.2 (0.18, 0.21) | 1.8%, 0.01 (0, 0.01)* |
| All | Respiratory season | Log score | Deaths | 37.4%, 0.16 (0.15, 0.17) | 41.1%, 0.17 (0.15, 0.18) | 2.64%, 0.01 (0, 0.01)* |
| All | Respiratory season | Accuracy | Cases | 60.3%, 20% (20%, 25%) | 75.3%, 25% (25%, 30%) | 9.38%, 5.01% (5%, 5%) |
| All | Respiratory season | Accuracy | Deaths | 47.3%, 25% (20%, 25%) | 54.3%, 25% (25%, 30%) | 4.76%, 5% (0%, 5%) |
| All | Off season | Log score | Cases | -25.9%, -0.14 (-0.14, -0.13)* | -24.6%, -0.14 (-0.16, -0.13)* | 1.68%, 0 (0, 0.01)* |
| All | Off season | Log score | Deaths | -1.3%, 0.02 (0.02, 0.03)* | -0.86%, 0.02 (0.02, 0.02)* | 0.444%, -0.0031 (-0.0036, -0.0025)* |
| All | Off season | Accuracy | Cases | -5.95%, -5% (-5%, 0%)* | -6.04%, 0% (-4.99%, 0%)* | -0.0987%, 0% (0%, 0.01%)* |
| All | Off season | Accuracy | Deaths | -6.77%, -5% (-5%, -4.99%)* | -7.64%, -4.99% (-5%, -5%)* | -0.94%, 0% (0%, 0.01%)* |
| 2nd wave | Off season | Log score | Cases | 32%, 0.22 (0.2, 0.24) | 23.5%, 0.16 (0.14, 0.17) | -6.42%, -0.056 (-0.063, -0.051)* |
| 2nd wave | Off season | Log score | Deaths | 23.9%, 0.17 (0.16, 0.19) | 17.1%, 0.13 (0.12, 0.14) | -5.5%, -0.048 (-0.052, -0.043)* |
| 2nd wave | Off season | Accuracy | Cases | 37.6%, 15% (15%, 20%) | 35.6%, 20% (15%, 20%) | -1.43%, -0.01% (-5%, 0%)* |
| 2nd wave | Off season | Accuracy | Deaths | 45.6%, 25% (20%, 30%) | 28.1%, 20% (15%, 20%) | -12%, -10% (-15%, -10%)* |
| Alpha | Off season | Log score | Cases | -9.35%, 0.01 (0, 0.01)* | -24.7%, -0.052 (-0.11, 0)* | -16.9%, -0.087 (-0.11, -0.052)* |
| Alpha | Off season | Log score | Deaths | -3.33%, 0.01 (0.01, 0.01)* | -10.8%, 0.01 (0.01, 0.02)* | -7.74%, 0 (0, 0)* |
| Alpha | Off season | Accuracy | Cases | -42.4%, -30% (-35%, -25%)* | -39.8%, -30% (-35%, -25%)* | 4.52%, 5% (-0.01%, 5.01%) |
| Alpha | Off season | Accuracy | Deaths | -28.5%, -25% (-25%, -20%)* | -33.8%, -30% (-30%, -25%)* | -7.53%, -5.01% (-10%, 0%)* |
| Delta | Off season | Log score | Cases | -49.6%, -0.57 (-0.61, -0.55)* | -35.4%, -0.32 (-0.35, -0.3)* | 28.2%, 0.25 (0.23, 0.26) |
| Delta | Off season | Log score | Deaths | -12%, 0.02 (0.02, 0.02)* | -6.52%, 0.02 (0.02, 0.02)* | 6.28%, -0.0024 (-0.003, -0.0015)* |
| Delta | Off season | Accuracy | Cases | -55.8%, -25% (-25%, -20%)* | -50%, -20% (-25%, -20%)* | 13.1%, 4.99% (0%, 5%)* |
| Delta | Off season | Accuracy | Deaths | -50.3%, -25% (-25%, -20%)* | -43.2%, -20% (-20%, -15%)* | 14.5%, 5% (4.99%, 10%) |
| Omicron | Off season | Log score | Cases | -19.5%, -0.15 (-0.16, -0.14)* | -24%, -0.16 (-0.17, -0.15)* | -5.68%, -0.013 (-0.017, -0.0085)* |
| Omicron | Off season | Log score | Deaths | 4.77%, 0.03 (0.03, 0.03)* | 3.84%, 0.02 (0.02, 0.03)* | -0.887%, -0.0035 (-0.0041, -0.003)* |
| Omicron | Off season | Accuracy | Cases | 4.46%, 0.01% (0%, 5%)* | 2.52%, 0% (0%, 5%)* | -1.86%, -0.01% (0%, 0%)* |
| Omicron | Off season | Accuracy | Deaths | -1.59%, 0% (0%, 0%)* | -1.78%, 0% (0%, 0%)* | -0.191%, -0.01% (0%, 0%)* |

**Table D.** Impact of seasonality, by state. Numbers show the relative difference in mean log score or point prediction accuracy, the median of pair-wise difference in log score (95% CI of the median); asterisk (*) indicates if the median is significantly >0 or <0 at the α = 0.05 level, per a Wilcoxon rank sum test. A positive difference indicates superior log score or point prediction accuracy of the first listed approach; a negative difference indicates superior log score or point prediction accuracy of the second listed approach.

| State | Season | Metric | Measure | Fixed vs no seasonality | Transformed vs no seasonality | Transformed vs fixed seasonality |
| --- | --- | --- | --- | --- | --- | --- |
| California | All | Log score | Cases | -7.14%, -0.042 (-0.062, -0.024)* | -1.53%, -0.028 (-0.045, -0.013)* | 6.04%, 0.02 (0.01, 0.02)* |
| California | All | Log score | Deaths | 16.7%, 0.04 (0.04, 0.05)* | 21.4%, 0.04 (0.03, 0.04)* | 3.96%, 0 (0, 0.01)* |
| California | All | Accuracy | Cases | 13.1%, 5% (0%, 9.99%) | 11.3%, 5% (0%, 10%) | -1.6%, 0% (0%, 0%)* |
| California | All | Accuracy | Deaths | 4.74%, 0% (0.01%, 5%)* | 1.86%, 0% (-0.01%, 5%)* | -2.75%, 0% (-4.99%, 0%)* |
| California | Respiratory season | Log score | Cases | 42.5%, 0.17 (0.13, 0.22) | 56.7%, 0.24 (0.19, 0.3) | 9.96%, 0.05 (0.04, 0.06)* |
| California | Respiratory season | Log score | Deaths | 48.4%, 0.21 (0.15, 0.3) | 60%, 0.27 (0.19, 0.37) | 7.8%, 0.03 (0.02, 0.04)* |
| California | Respiratory season | Accuracy | Cases | 51.9%, 20% (15%, 20%) | 58.9%, 20% (15%, 25%) | 4.61%, 4.99% (-0.01%, 5%)* |
| California | Respiratory season | Accuracy | Deaths | 27.9%, 15% (10%, 20%) | 22.4%, 10% (5%, 15%) | -4.33%, -5% (-10%, 0%)* |
| California | Off season | Log score | Cases | -30.3%, -0.17 (-0.2, -0.14)* | -27.9%, -0.19 (-0.23, -0.16)* | 3.49%, -0.0024 (-0.013, 0)* |
| California | Off season | Log score | Deaths | -0.598%, 0.03 (0.02, 0.03)* | 0.856%, 0.02 (0.02, 0.03)* | 1.46%, -1e-05 (-0.0014, 0.00098)* |
| California | Off season | Accuracy | Cases | -11%, -5% (-10%, 0%)* | -18.4%, -9.99% (-15%, -5.01%)* | -8.21%, -5% (-10%, 0%)* |
| California | Off season | Accuracy | Deaths | -8.26%, -5% (-10%, 0%)* | -9.65%, -5% (-10%, -5%)* | -1.52%, 0% (-4.99%, 5%)* |
| Florida | All | Log score | Cases | -12.6%, -0.02 (-0.032, -0.01)* | -6.11%, 0.001 (-0.0085, 0.01)* | 7.47%, 0.03 (0.02, 0.04)* |
| Florida | All | Log score | Deaths | 0.697%, 0.02 (0.01, 0.02)* | 4.42%, 0.02 (0.02, 0.03)* | 3.7%, 0.01 (0.01, 0.01)* |
| Florida | All | Accuracy | Cases | 8.52%, 5% (5%, 10%) | 11.5%, 5% (5%, 10%) | 2.74%, 0% (0%, 5%)* |
| Florida | All | Accuracy | Deaths | 3.73%, 5% (0%, 5.01%) | 4.9%, 5% (0%, 5.01%) | 1.14%, 0% (0%, 0%)* |
| Florida | Respiratory season | Log score | Cases | -2.01%, 0.03 (0.02, 0.05)* | 1.72%, 0.04 (0.02, 0.05)* | 3.81%, 0.02 (0.01, 0.04)* |
| Florida | Respiratory season | Log score | Deaths | 2.77%, 0.05 (0.04, 0.06) | 8.52%, 0.06 (0.04, 0.07) | 5.59%, 0.03 (0.02, 0.04)* |
| Florida | Respiratory season | Accuracy | Cases | 19%, 10% (9.99%, 15%) | 20.7%, 15% (10%, 20%) | 1.44%, 0% (-5%, 5%)* |
| Florida | Respiratory season | Accuracy | Deaths | 11.8%, 10% (9.99%, 15%) | 12.9%, 10% (5%, 15%) | 0.958%, 0% (-5%, 4.99%)* |
| Florida | Off season | Log score | Cases | -19.1%, -0.066 (-0.088, -0.046)* | -11%, -0.02 (-0.037, -0.0059)* | 10%, 0.03 (0.02, 0.05)* |
| Florida | Off season | Log score | Deaths | -0.675%, 0.01 (0.01, 0.01)* | 1.75%, 0.01 (0.01, 0.02)* | 2.44%, 0 (0.00049, 0)* |
| Florida | Off season | Accuracy | Cases | 2.1%, 0% (0%, 5%)* | 5.84%, 5% (0%, 5%) | 3.66%, 0% (0.01%, 5%)* |
| Florida | Off season | Accuracy | Deaths | -1.82%, 0% (-5%, 0%)* | -0.565%, 0% (0%, 4.99%)* | 1.28%, 0.01% (0%, 4.99%)* |
| Iowa | All | Log score | Cases | 1.02%, 0.02 (0.01, 0.03)* | 6.96%, 0.03 (0.02, 0.04)* | 5.89%, 0.02 (0.01, 0.03)* |
| Iowa | All | Log score | Deaths | 23.6%, 0.07 (0.06, 0.08) | 25.7%, 0.06 (0.05, 0.07) | 1.64%, -0.001 (-0.0031, 5e-04)* |
| Iowa | All | Accuracy | Cases | 12.9%, 5% (0%, 10%) | 28.3%, 10% (10%, 15%) | 13.7%, 10% (5%, 10%) |
| Iowa | All | Accuracy | Deaths | 17.8%, 10% (5%, 10%) | 22.7%, 10% (10%, 15%) | 4.19%, 5% (0.01%, 5.01%) |
| Iowa | Respiratory season | Log score | Cases | 77.8%, 0.47 (0.4, 0.53) | 77.4%, 0.48 (0.42, 0.54) | -0.246%, 0.01 (-0.003, 0.02)* |
| Iowa | Respiratory season | Log score | Deaths | 72.9%, 0.38 (0.31, 0.46) | 76.2%, 0.44 (0.36, 0.5) | 1.95%, 0.01 (-0.001, 0.02)* |
| Iowa | Respiratory season | Accuracy | Cases | 71.5%, 25% (20%, 25%) | 112%, 35% (30%, 40%) | 23.4%, 15% (9.99%, 15%) |
| Iowa | Respiratory season | Accuracy | Deaths | 81%, 35% (30%, 35%) | 88.7%, 35% (30%, 40%) | 4.26%, 5% (0.01%, 10%) |
| Iowa | Off season | Log score | Cases | -30.2%, -0.11 (-0.14, -0.076)* | -23.2%, -0.076 (-0.1, -0.05)* | 10.1%, 0.02 (0.01, 0.03)* |
| Iowa | Off season | Log score | Deaths | -0.723%, 0.03 (0.03, 0.04)* | 0.71%, 0.03 (0.03, 0.03)* | 1.44%, -0.0035 (-0.0055, -0.0025)* |
| Iowa | Off season | Accuracy | Cases | -17.6%, -10% (-15%, -5%)* | -14.9%, -10% (-10%, -4.99%)* | 3.29%, 0% (0%, 5%)* |
| Iowa | Off season | Accuracy | Deaths | -18.8%, -10% (-15%, -10%)* | -15.5%, -10% (-15%, -5.01%)* | 4.1%, 0% (0%, 5%)* |
| Massachusetts | All | Log score | Cases | 8.31%, 0.05 (0.03, 0.07) | 4.64%, 0.02 (0, 0.03)* | -3.39%, -0.03 (-0.038, -0.023)* |
| Massachusetts | All | Log score | Deaths | 30%, 0.11 (0.1, 0.12) | 28.8%, 0.08 (0.07, 0.09) | -0.937%, -0.015 (-0.018, -0.012)* |
| Massachusetts | All | Accuracy | Cases | 49.1%, 20% (15%, 25%) | 33.5%, 15% (10%, 15%) | -10.4%, -10% (-10%, -5%)* |
| Massachusetts | All | Accuracy | Deaths | 34.7%, 20% (15%, 20%) | 28.1%, 15% (10%, 20%) | -4.87%, -5% (-5%, 0%)* |
| Massachusetts | Respiratory season | Log score | Cases | 83.6%, 0.5 (0.44, 0.57) | 78%, 0.45 (0.39, 0.52) | -3.05%, -0.034 (-0.044, -0.023)* |
| Massachusetts | Respiratory season | Log score | Deaths | 72.7%, 0.42 (0.34, 0.49) | 71%, 0.41 (0.35, 0.48) | -0.976%, -0.021 (-0.028, -0.015)* |
| Massachusetts | Respiratory season | Accuracy | Cases | 121%, 35% (35%, 40%) | 99.7%, 35% (30%, 40%) | -9.73%, -10% (-10%, -5%)* |
| Massachusetts | Respiratory season | Accuracy | Deaths | 68.3%, 30% (25%, 35%) | 56.7%, 25% (25%, 30%) | -6.9%, -5% (-10%, -5%)* |
| Massachusetts | Off season | Log score | Cases | -24%, -0.074 (-0.11, -0.046)* | -26.8%, -0.18 (-0.24, -0.14)* | -3.62%, -0.026 (-0.036, -0.017)* |
| Massachusetts | Off season | Log score | Deaths | 7.39%, 0.05 (0.04, 0.06)* | 6.42%, 0.03 (0.03, 0.04)* | -0.91%, -0.012 (-0.014, -0.0096)* |
| Massachusetts | Off season | Accuracy | Cases | 3.66%, 0% (0%, 5%)* | -8.13%, -4.99% (-10%, 0%)* | -11.4%, -5% (-10%, -5%)* |
| Massachusetts | Off season | Accuracy | Deaths | 7.54%, 5% (0.01%, 9.99%) | 5.05%, 5% (0%, 5%) | -2.31%, 0% (-5%, 0%)* |
| Michigan | All | Log score | Cases | 0.571%, -0.0055 (-0.024, 0.01)* | -9.27%, -0.034 (-0.054, -0.015)* | -9.79%, -0.03 (-0.038, -0.023)* |
| Michigan | All | Log score | Deaths | 16.8%, 0.06 (0.05, 0.08) | 7.52%, 0.04 (0.03, 0.05)* | -7.95%, -0.032 (-0.036, -0.028)* |
| Michigan | All | Accuracy | Cases | 26.8%, 10% (9.99%, 15%) | 20.5%, 10% (5%, 10%) | -5.01%, -5% (-5%, 0%)* |
| Michigan | All | Accuracy | Deaths | 21.6%, 10% (9.99%, 15%) | 10.2%, 5% (5%, 9.99%) | -9.42%, -5% (-9.99%, -5%)* |
| Michigan | Respiratory season | Log score | Cases | 38.8%, 0.24 (0.19, 0.29) | 29.1%, 0.19 (0.15, 0.24) | -7.03%, -0.036 (-0.044, -0.027)* |
| Michigan | Respiratory season | Log score | Deaths | 38.7%, 0.19 (0.15, 0.24) | 33%, 0.16 (0.12, 0.2) | -4.09%, -0.036 (-0.043, -0.028)* |
| Michigan | Respiratory season | Accuracy | Cases | 116%, 35% (30%, 40%) | 84.3%, 25% (25%, 30%) | -14.6%, -10% (-15%, -5%)* |
| Michigan | Respiratory season | Accuracy | Deaths | 83.9%, 40% (35%, 40%) | 44.1%, 25% (20%, 25%) | -21.6%, -20% (-20%, -15%)* |
| Michigan | Off season | Log score | Cases | -18.6%, -0.14 (-0.18, -0.11)* | -28%, -0.21 (-0.24, -0.16)* | -11.6%, -0.029 (-0.042, -0.017)* |
| Michigan | Off season | Log score | Deaths | 4.38%, 0.04 (0.03, 0.05)* | -6.47%, 0.02 (0.01, 0.03)* | -10.4%, -0.028 (-0.032, -0.024)* |
| Michigan | Off season | Accuracy | Cases | -16.4%, -10% (-10%, -5%)* | -10.6%, -5.01% (-9.99%, 0%)* | 7.01%, 5% (0%, 10%) |
| Michigan | Off season | Accuracy | Deaths | -18%, -10% (-15%, -10%)* | -11.4%, -5% (-10%, 0%)* | 8.01%, 5.01% (0%, 10%) |
| New York | All | Log score | Cases | -3.26%, 0.01 (5.9e-06, 0.02)* | -4.56%, -0.002 (-0.02, 0.01)* | -1.34%, -0.0065 (-0.014, 6.4e-05)* |
| New York | All | Log score | Deaths | 19%, 0.08 (0.07, 0.09) | 17.9%, 0.06 (0.05, 0.07) | -0.852%, -0.011 (-0.014, -0.0086)* |
| New York | All | Accuracy | Cases | 30.7%, 15% (10%, 15%) | 51%, 20% (20%, 25%) | 15.6%, 10% (5%, 15%) |
| New York | All | Accuracy | Deaths | 21.7%, 10% (10%, 15%) | 32.1%, 20% (15%, 20%) | 8.58%, 10% (5%, 10%) |
| New York | Respiratory season | Log score | Cases | 44.9%, 0.24 (0.21, 0.28) | 44.3%, 0.24 (0.2, 0.28) | -0.435%, -0.007 (-0.019, 0)* |
| New York | Respiratory season | Log score | Deaths | 37.8%, 0.21 (0.18, 0.25) | 37.8%, 0.21 (0.17, 0.24) | -0.00206%, -0.0056 (-0.014, 0)* |
| New York | Respiratory season | Accuracy | Cases | 62.7%, 25% (20%, 30%) | 100%, 35% (30%, 40%) | 22.9%, 15% (10%, 20%) |
| New York | Respiratory season | Accuracy | Deaths | 51.3%, 25% (20%, 30%) | 80.5%, 40% (35%, 40%) | 19.3%, 15% (15%, 20%) |
| New York | Off season | Log score | Cases | -26.3%, -0.11 (-0.14, -0.085)* | -27.7%, -0.2 (-0.25, -0.15)* | -1.94%, -0.0074 (-0.017, 2.2e-05)* |
| New York | Off season | Log score | Deaths | 7.78%, 0.05 (0.05, 0.06) | 6.25%, 0.04 (0.03, 0.04)* | -1.42%, -0.012 (-0.014, -0.01)* |
| New York | Off season | Accuracy | Cases | 6.51%, 5% (-0.01%, 10%) | 14%, 5% (0%, 10%) | 7.02%, 5% (0%, 10%) |
| New York | Off season | Accuracy | Deaths | -0.706%, -0.01% (-4.99%, 5%)* | -4.44%, -5% (-10%, 0%)* | -3.76%, -5% (-5%, 0%)* |
| Pennsylvania | All | Log score | Cases | -5.3%, -0.008 (-0.026, 0.01)* | -5.72%, 0.01 (-0.0085, 0.02)* | -0.438%, -0.0039 (-0.011, 0)* |
| Pennsylvania | All | Log score | Deaths | 13.8%, 0.07 (0.06, 0.08) | 12.9%, 0.06 (0.05, 0.07) | -0.791%, -0.012 (-0.016, -0.009)* |
| Pennsylvania | All | Accuracy | Cases | 29.7%, 10% (10%, 15%) | 47.5%, 20% (15%, 20%) | 13.7%, 10% (5%, 10%) |
| Pennsylvania | All | Accuracy | Deaths | 29.4%, 15% (10%, 20%) | 36.4%, 20% (15%, 20%) | 5.39%, 5% (0%, 9.99%) |
| Pennsylvania | Respiratory season | Log score | Cases | 38.5%, 0.18 (0.14, 0.22) | 38.6%, 0.2 (0.17, 0.23) | 0.0339%, -0.003 (-0.016, 0.01)* |
| Pennsylvania | Respiratory season | Log score | Deaths | 36.2%, 0.15 (0.12, 0.18) | 35.4%, 0.14 (0.11, 0.16) | -0.636%, -0.017 (-0.028, -0.006)* |
| Pennsylvania | Respiratory season | Accuracy | Cases | 48.9%, 20% (15%, 25%) | 99.6%, 35% (30%, 40%) | 34.1%, 20% (15%, 25%) |
| Pennsylvania | Respiratory season | Accuracy | Deaths | 50.6%, 25% (20%, 30%) | 76.7%, 40% (35%, 40%) | 17.4%, 15% (10%, 20%) |
| Pennsylvania | Off season | Log score | Cases | -26.7%, -0.18 (-0.23, -0.14)* | -27.2%, -0.19 (-0.24, -0.14)* | -0.754%, -0.0046 (-0.012, 0)* |
| Pennsylvania | Off season | Log score | Deaths | 0.845%, 0.05 (0.04, 0.06)* | -0.0572%, 0.04 (0.04, 0.05)* | -0.895%, -0.011 (-0.014, -0.0085)* |
| Pennsylvania | Off season | Accuracy | Cases | 13%, 5% (0.01%, 9.99%) | 2.05%, 0% (-0.01%, 4.99%)* | -9.71%, -5.01% (-10%, -4.99%)* |
| Pennsylvania | Off season | Accuracy | Deaths | 11.1%, 5% (0%, 10%) | 1.46%, 0% (-4.99%, 5%)* | -8.66%, -5% (-10%, -5%)* |
| Texas | All | Log score | Cases | -9.81%, -0.02 (-0.043, -0.00054)* | 1.82%, 0.01 (-0.0066, 0.02)* | 12.9%, 0.06 (0.05, 0.07) |
| Texas | All | Log score | Deaths | 6.34%, 0.03 (0.02, 0.03)* | 15.6%, 0.03 (0.02, 0.03)* | 8.68%, 0.01 (0.01, 0.01)* |
| Texas | All | Accuracy | Cases | 11.1%, 5% (0.01%, 5%) | 33.5%, 15% (10%, 15%) | 20.2%, 10% (10%, 15%) |
| Texas | All | Accuracy | Deaths | 9.13%, 5% (0%, 5%) | 27.6%, 15% (10%, 15%) | 16.9%, 10% (9.99%, 15%) |
| Texas | Respiratory season | Log score | Cases | 59.4%, 0.34 (0.26, 0.42) | 77.3%, 0.43 (0.36, 0.51) | 11.3%, 0.05 (0.04, 0.07) |
| Texas | Respiratory season | Log score | Deaths | 51.9%, 0.3 (0.23, 0.37) | 68%, 0.42 (0.35, 0.49) | 10.6%, 0.07 (0.05, 0.08) |
| Texas | Respiratory season | Accuracy | Cases | 50.1%, 20% (15%, 25%) | 96.9%, 30% (25%, 35%) | 31.2%, 20% (15%, 25%) |
| Texas | Respiratory season | Accuracy | Deaths | 47%, 25% (20%, 25%) | 91.5%, 40% (35%, 40%) | 30.2%, 25% (20%, 25%) |
| Texas | Off season | Log score | Cases | -38.5%, -0.22 (-0.27, -0.18)* | -29.9%, -0.13 (-0.17, -0.096)* | 14%, 0.06 (0.05, 0.08) |
| Texas | Off season | Log score | Deaths | -16.3%, 0.02 (0.02, 0.02)* | -10.1%, 0.01 (0.01, 0.02)* | 7.43%, -1.8e-06 (-0.00099, 0.00049)* |
| Texas | Off season | Accuracy | Cases | -16%, -10% (-15%, -5.01%)* | -10.5%, -5% (-10%, -5%)* | 6.57%, 5% (0.01%, 5%) |
| Texas | Off season | Accuracy | Deaths | -18.8%, -15% (-15%, -10%)* | -19.6%, -15% (-15%, -10%)* | -0.909%, 0% (-0.01%, 0%)* |
| Washington | All | Log score | Cases | -14%, -0.092 (-0.11, -0.078)* | -13.5%, -0.098 (-0.11, -0.084)* | 0.577%, 0.01 (0, 0.01)* |
| Washington | All | Log score | Deaths | -4.44%, -0.002 (-0.007, 0.00092)* | -1.65%, 0 (-0.0015, 0)* | 2.92%, 0.01 (0.01, 0.01)* |
| Washington | All | Accuracy | Cases | 16.5%, 5% (4.99%, 10%) | 13.5%, 5% (0.01%, 10%) | -2.54%, 0% (0%, 0.01%)* |
| Washington | All | Accuracy | Deaths | 11%, 5.01% (0%, 10%) | 11.3%, 5.01% (5%, 9.99%) | 0.291%, 0% (-0.01%, 0%)* |
| Washington | Respiratory season | Log score | Cases | 0.837%, -0.026 (-0.046, -0.0079)* | 3.75%, -0.0025 (-0.018, 0.01)* | 2.89%, 0.03 (0.02, 0.03)* |
| Washington | Respiratory season | Log score | Deaths | 0.291%, -0.05 (-0.068, -0.032)* | 2.95%, -0.032 (-0.046, -0.017)* | 2.65%, 0.02 (0.01, 0.02)* |
| Washington | Respiratory season | Accuracy | Cases | 27%, 10% (5%, 15%) | 25.6%, 10% (5.01%, 15%) | -1.03%, 0% (-0.01%, 0%)* |
| Washington | Respiratory season | Accuracy | Deaths | 23.9%, 10% (10%, 15%) | 29.2%, 15% (10%, 20%) | 4.31%, 4.99% (0%, 5%)* |
| Washington | Off season | Log score | Cases | -22.7%, -0.15 (-0.17, -0.13)* | -23.4%, -0.19 (-0.22, -0.17)* | -0.949%, -0.01 (-0.018, -0.0035)* |
| Washington | Off season | Log score | Deaths | -7.49%, 0.01 (0.01, 0.01)* | -4.62%, 0.01 (0.01, 0.01)* | 3.1%, 0 (0, 0)* |
| Washington | Off season | Accuracy | Cases | 5.51%, 0.01% (0%, 5.01%)* | 0.819%, 0.01% (0%, 5%)* | -4.45%, 0% (-5%, 0%)* |
| Washington | Off season | Accuracy | Deaths | -1.29%, -0.01% (-5%, 0%)* | -5.72%, -5% (-5%, 0%)* | -4.5%, -5% (-5%, 0%)* |
| Wyoming | All | Log score | Cases | 0.44%, -0.00092 (-0.007, 0)* | 1.31%, 0 (-0.0045, 0.01)* | 0.869%, 0.01 (0.01, 0.01)* |
| Wyoming | All | Log score | Deaths | 8.84%, 0.03 (0.02, 0.03)* | 12.6%, 0.03 (0.02, 0.03)* | 3.47%, 0 (0, 0.01)* |
| Wyoming | All | Accuracy | Cases | 6.6%, 4.99% (0%, 5%)* | 14.4%, 5% (5%, 10%) | 7.28%, 5% (0.01%, 5.01%) |
| Wyoming | All | Accuracy | Deaths | 3.37%, 0.01% (0%, 5%)* | 7.98%, 5% (0%, 5%) | 4.46%, 0% (-0.01%, 5%)* |
| Wyoming | Respiratory season | Log score | Cases | 40.7%, 0.19 (0.16, 0.22) | 42.9%, 0.17 (0.15, 0.2) | 1.51%, -0.0036 (-0.013, 0)* |
| Wyoming | Respiratory season | Log score | Deaths | 34.1%, 0.18 (0.14, 0.21) | 39.6%, 0.16 (0.13, 0.19) | 4.1%, 8.3e-05 (-0.0084, 0.01)* |
| Wyoming | Respiratory season | Accuracy | Cases | 57.9%, 20% (15%, 25%) | 70.8%, 25% (20%, 30%) | 8.14%, 5% (0.01%, 10%) |
| Wyoming | Respiratory season | Accuracy | Deaths | 29.6%, 15% (10%, 20%) | 41.1%, 20% (15%, 25%) | 8.87%, 5% (0%, 10%) |
| Wyoming | Off season | Log score | Cases | -19.5%, -0.15 (-0.18, -0.12)* | -19.1%, -0.13 (-0.16, -0.093)* | 0.454%, 0.02 (0.01, 0.02)* |
| Wyoming | Off season | Log score | Deaths | -5.06%, 0.01 (0.01, 0.01)* | -2.16%, 0.01 (0.01, 0.02)* | 3.05%, 0.01 (0, 0.01)* |
| Wyoming | Off season | Accuracy | Cases | -20%, -10% (-15%, -5%)* | -14.9%, -5% (-10%, 0%)* | 6.39%, 5% (0%, 10%) |
| Wyoming | Off season | Accuracy | Deaths | -14.2%, -9.99% (-10%, -5.01%)* | -14.2%, -9.99% (-10%, -5%)* | 0%, 0% (-0.01%, 5%)* |

**Table E.** Comparison of forecast performance of the ARIMAX models. Only four models (see the top row for model names) are shown here because the fifth model (ARIMAX.FULL with vaccination included) was only able to generate forecasts for less than half of the study weeks; see details on the models in the main text. Numbers show the mean log score or point prediction accuracy of forecasts (specified in the “metric” column), aggregated across the entire study period and all locations for all forecast targets combined or individual forecast targets (specified in the “target” column). Bolded fonts indicate best performance (highest log score or accuracy).

| target | metric | measure | Models | | | |
| --- | --- | --- | --- | --- | --- | --- |
|  |  |  | ARIMA | ARIMAX.MOB | ARIMAX.SN | ARIMAX.MS |
| all | Log score | Cases | -2.64 | **-2.53** | -2.75 | -3.03 |
| all | Log score | Deaths | -1.77 | -1.73 | **-1.64** | -1.71 |
| all | Accuracy | Cases | 13% | 15% | **18%** | 15% |
| all | Accuracy | Deaths | 14% | 17% | **21%** | 17% |
| 1-8wk ahead | Log score | Cases | -2.08 | **-2** | -2.04 | -2.08 |
| 1-8wk ahead | Log score | Deaths | -1.34 | -1.36 | **-1.27** | -1.34 |
| 1-8wk ahead | Accuracy | Cases | 22% | 25% | **26%** | 24% |
| 1-8wk ahead | Accuracy | Deaths | 22% | 25% | **28%** | 23% |
| 9-16wk ahead | Log score | Cases | -2.86 | **-2.62** | -2.8 | -3.14 |
| 9-16wk ahead | Log score | Deaths | -1.89 | -1.8 | **-1.64** | -1.68 |
| 9-16wk ahead | Accuracy | Cases | 8% | 11% | **14%** | 11% |
| 9-16wk ahead | Accuracy | Deaths | 8% | 12% | **18%** | 12% |
| 17-26wk ahead | Log score | Cases | -2.89 | **-2.77** | -3.26 | -3.77 |
| 17-26wk ahead | Log score | Deaths | -1.82 | -1.75 | **-1.72** | -1.76 |
| 17-26wk ahead | Accuracy | Cases | 8% | 10% | **12%** | 10% |
| 17-26wk ahead | Accuracy | Deaths | 10% | 12% | **16%** | 12% |
| peak intensity | Log score | Cases | **-3.87** | -4.45 | -4.39 | -4.57 |
| peak intensity | Log score | Deaths | **-3.14** | -3.38 | -3.18 | -3.5 |
| peak intensity | Accuracy | Cases | 14% | 15% | **19%** | 18% |
| peak intensity | Accuracy | Deaths | 15% | 18% | **23%** | 21% |
| peak week | Log score | Cases | **-2.63** | -2.66 | -3.01 | -3.21 |
| peak week | Log score | Deaths | -2.54 | -2.53 | **-2.36** | -2.45 |
| peak week | Accuracy | Cases | 11% | 12% | **20%** | 21% |
| peak week | Accuracy | Deaths | 11% | 13% | **23%** | 23% |
| total | Log score | Cases | -2.37 | **-2.36** | -2.54 | -2.5 |
| total | Log score | Deaths | **-2.22** | -2.27 | -2.29 | -2.56 |
| total | Accuracy | Cases | 10% | 12% | **13%** | **13%** |
| total | Accuracy | Deaths | 12% | 13% | 17% | **19%** |

**Table F.** Comparison of forecast performance of the approaches developed in this study with the best-performing ARIMAX model. Numbers show the mean log score or point prediction accuracy of forecasts (specified in the “metric” column), aggregated across the entire study period and all locations for all forecast targets combined or individual forecast targets (specified in the “target” column). Bolded fonts indicate best performance (highest log score or accuracy).

|  |  | Log score |  |  | Accuracy |  |  |
| --- | --- | --- | --- | --- | --- | --- | --- |
| target | measure | ARIMAX.SN | Baseline | Best-performing | ARIMAX.SN | Baseline | Best-performing |
| all | Cases | -2.75 | -1.95 | **-1.46** | 18% | 11% | **26%** |
| all | Deaths | -1.64 | -0.97 | **-0.65** | 21% | 17% | **31%** |
| 1-8wk ahead | Cases | -2.04 | -1.08 | **-0.91** | 26% | 26% | **38%** |
| 1-8wk ahead | Deaths | -1.27 | -0.42 | **-0.35** | 28% | 39% | **48%** |
| 9-16wk ahead | Cases | -2.8 | -1.86 | **-1.49** | 14% | 4% | **20%** |
| 9-16wk ahead | Deaths | -1.64 | -0.83 | **-0.64** | 18% | 7% | **25%** |
| 17-26wk ahead | Cases | -3.26 | -2.8 | **-1.87** | 12% | 1% | **16%** |
| 17-26wk ahead | Deaths | -1.72 | -1.43 | **-0.8** | **16%** | 1% | **16%** |
| peak intensity | Cases | -4.39 | -2.43 | **-2.01** | 19% | 20% | **40%** |
| peak intensity | Deaths | -3.18 | -1.69 | **-1.36** | 23% | 30% | **51%** |
| peak week | Cases | -3.01 | -3.51 | **-2.73** | 20% | 24% | **42%** |
| peak week | Deaths | -2.36 | -2.61 | **-1.53** | 23% | 35% | **56%** |
| total | Cases | -2.54 | -1.05 | **-0.75** | 13% | 7% | **33%** |
| total | Deaths | -2.29 | -0.99 | **-0.67** | 17% | 9% | **36%** |

**Table G.** Preliminary assessment of the real-time forecasts initiated the week of October 2, 2022 for October 2022 – March 2023. The log score and accuracy were computed using reported case and mortality data downloaded on March 31, 2023 (see further details in the main text). As shown in Fig 8, COVID-19 mortality data in some states (e.g., Wyoming) were highly irregular during the forecast period, likely an artifact of reporting. Due to these potential data inaccuracies, the mortality-related log score and point prediction accuracy for these states are likely lower than the true values (to be obtained once more complete mortality data are available).

| State | target | Log score |  | Accuracy |  |
| --- | --- | --- | --- | --- | --- |
|  |  | Cases | Deaths | Cases | Deaths |
| All | all | -0.45 | -0.2 | 56% | 23% |
| All | 1-8wk ahead | -0.31 | -0.06 | 56% | 22% |
| All | 9-16wk ahead | -0.71 | -0.17 | 46% | 20% |
| All | 17-26wk ahead | -0.25 | -0.12 | 64% | 28% |
| All | peak intensity | -0.84 | -0.32 | 48% | 6% |
| All | peak week | -1.21 | -2.3 | 52% | 41% |
| All | total | -0.21 | -0.13 | 78% | 7% |
| California | all | -0.52 | -0.1 | 60% | 52% |
| California | 1-8wk ahead | -0.31 | -0.04 | 55% | 32% |
| California | 9-16wk ahead | -0.99 | -0.11 | 41% | 74% |
| California | 17-26wk ahead | -0.24 | -0.06 | 73% | 43% |
| California | peak intensity | -1.39 | -0.1 | 90% | 60% |
| California | peak week | -0.72 | -0.86 | 100% | 100% |
| California | total | -0.24 | -0.1 | 60% | 60% |
| Florida | all | -0.38 | -0.19 | 28% | 8% |
| Florida | 1-8wk ahead | -0.06 | -0.02 | 5% | 0% |
| Florida | 9-16wk ahead | -0.66 | -0.2 | 9% | 14% |
| Florida | 17-26wk ahead | -0.31 | -0.16 | 56% | 13% |
| Florida | peak intensity | -1.2 | -0.54 | 0% | 0% |
| Florida | peak week | -0.74 | -1.6 | 90% | 0% |
| Florida | total | -0.16 | -0.05 | 50% | 0% |
| Iowa | all | -0.37 | -0.12 | 53% | 29% |
| Iowa | 1-8wk ahead | -0.2 | -0.05 | 86% | 12% |
| Iowa | 9-16wk ahead | -0.59 | -0.09 | 42% | 24% |
| Iowa | 17-26wk ahead | -0.2 | -0.07 | 29% | 49% |
| Iowa | peak intensity | -0.71 | -0.11 | 80% | 0% |
| Iowa | peak week | -1.37 | -1.38 | 60% | 70% |
| Iowa | total | -0.26 | -0.05 | 70% | 0% |
| Massachusetts | all | -0.64 | -0.31 | 61% | 9% |
| Massachusetts | 1-8wk ahead | -0.7 | -0.06 | 41% | 18% |
| Massachusetts | 9-16wk ahead | -0.86 | -0.46 | 72% | 6% |
| Massachusetts | 17-26wk ahead | -0.36 | -0.2 | 78% | 0% |
| Massachusetts | peak intensity | -1.33 | -0.61 | 0% | 0% |
| Massachusetts | peak week | -0.95 | -1.62 | 0% | 70% |
| Massachusetts | total | -0.26 | -0.7 | 80% | 0% |
| Michigan | all | -0.32 | -0.32 | 51% | 17% |
| Michigan | 1-8wk ahead | -0.3 | -0.2 | 45% | 25% |
| Michigan | 9-16wk ahead | -0.46 | -0.21 | 64% | 12% |
| Michigan | 17-26wk ahead | -0.11 | -0.22 | 41% | 19% |
| Michigan | peak intensity | -0.35 | -0.12 | 100% | 0% |
| Michigan | peak week | -1.81 | -3.59 | 0% | 0% |
| Michigan | total | -0.06 | -0.05 | 90% | 0% |
| New York | all | -0.76 | -0.14 | 66% | 17% |
| New York | 1-8wk ahead | -0.68 | -0.07 | 52% | 14% |
| New York | 9-16wk ahead | -1.27 | -0.05 | 48% | 0% |
| New York | 17-26wk ahead | -0.34 | -0.05 | 98% | 37% |
| New York | peak intensity | -0.97 | -0.11 | 30% | 0% |
| New York | peak week | -1.76 | -2.31 | 10% | 0% |
| New York | total | -0.24 | -0.06 | 90% | 0% |
| Pennsylvania | all | -0.49 | -0.12 | 59% | 37% |
| Pennsylvania | 1-8wk ahead | -0.33 | -0.05 | 81% | 25% |
| Pennsylvania | 9-16wk ahead | -0.75 | -0.1 | 34% | 30% |
| Pennsylvania | 17-26wk ahead | -0.34 | -0.08 | 62% | 53% |
| Pennsylvania | peak intensity | -0.73 | -0.16 | 0% | 0% |
| Pennsylvania | peak week | -1.09 | -1.33 | 100% | 90% |
| Pennsylvania | total | -0.21 | -0.08 | 80% | 0% |
| Texas | all | -0.37 | -0.08 | 64% | 10% |
| Texas | 1-8wk ahead | -0.22 | -0.03 | 78% | 4% |
| Texas | 9-16wk ahead | -0.67 | -0.05 | 60% | 19% |
| Texas | 17-26wk ahead | -0.18 | -0.04 | 50% | 3% |
| Texas | peak intensity | -0.75 | -0.09 | 70% | 0% |
| Texas | peak week | -0.83 | -1.22 | 100% | 70% |
| Texas | total | -0.26 | -0.05 | 80% | 0% |
| Washington | all | -0.26 | -0.3 | 78% | 31% |
| Washington | 1-8wk ahead | -0.21 | -0.04 | 75% | 65% |
| Washington | 9-16wk ahead | -0.35 | -0.1 | 62% | 12% |
| Washington | 17-26wk ahead | -0.15 | -0.07 | 92% | 26% |
| Washington | peak intensity | -0.52 | -0.08 | 90% | 0% |
| Washington | peak week | -0.76 | -6.85 | 60% | 0% |
| Washington | total | -0.22 | -0.06 | 90% | 10% |
| Wyoming | all | -0.35 | -0.29 | 44% | 21% |
| Wyoming | 1-8wk ahead | -0.14 | -0.03 | 40% | 25% |
| Wyoming | 9-16wk ahead | -0.47 | -0.3 | 29% | 9% |
| Wyoming | 17-26wk ahead | -0.27 | -0.21 | 63% | 33% |
| Wyoming | peak intensity | -0.42 | -1.29 | 20% | 0% |
| Wyoming | peak week | -2.1 | -2.23 | 0% | 10% |
| Wyoming | total | -0.21 | -0.04 | 90% | 0% |

**Table H.** Prior ranges for the parameters and variables used in the model-inference system. Parameters/state variables are initialized by drawing from uniform distributions specified in the rows labeled “Initialization”. During the filtering process, space-reprobing is applied to explore the state space, i.e., a small fraction of the ensemble members are randomly replaced with values drawn from the uniform distributions specified in the rows labeled with “Space-reprobing”.

| **Type** | **Parameters/ variables** | **Symbol** | **Range** | **Note** |
| --- | --- | --- | --- | --- |
| Initialization | Initial susceptible | S(t=0) | All locations: non-Omicron period, U[99%, 100%] population; Omicron period, U[50%, 90%] population | n/a |
| Initialization | Initial exposed | E(t=0) | All locations: U[5, 50] × no. cases during 1st week | n/a |
| Initialization | Initial infectious | I(t=0) | All locations: U[5,50] × no. cases during 1st week | n/a |
| Initialization | Infectious period | D | All locations: U[2, 5] days | n/a |
| Initialization | Latency period | Z | All locations: U[2, 5] days | n/a |
| Initialization | Duration of immunity (from prior infection) | L | All locations: non-Omicron period, U[2, 3] years; Omicron period: U[1, 3] years | n/a |
| Initialization | Time-to-detection, mean | Td, mean | All locations: U[5, 8] days | n/a |
| Initialization | Time-to-detection, sd | Td, sd | All locations: U[1, 3] days | To allow variation in time to diagnosis/reporting |
| Initialization | Scaling of NPI effectiveness | e | All locations: U[0.5, 1.5] | Around 1, with a large bound to be flexible |
| Initialization | Vaccine efficacy (VE) | n/a | All locations: before Delta, VE1=85%, VE2 = 95%; Delta, VE1 = 50%, VE2 = 80%; Omicron, VE1 = 10%, VE2 (combined 2nd and 3rd doses) = 70% | Used higher VE values, as the observations included both cases/infections and deaths; i.e., here VE is for both infections and mortality |
| Initialization | VE waning | *ρ* | rho(t) = 1/(1+exp(-k * (t - tm.imm/2); for wildtype: k = 0.026; tm.imm = 322; for Delta: k = 0.025; tm.imm = 280; for Omicron: k = 0.024; tm.imm = 256 | Parameter in the logistic function fitted based on data from UKHSA |
| Initialization | Infection-detection rate | *r* | all locations: U [0.01, 0.05] | n/a |
| Initialization | Infection-fatality risk | IFR | all locations: U [0.005, 0.015] | n/a |
| Initialization | Transmission rate | β | Wyoming: U [0.41, 0.6]; Iowa, Texas, Washington: U [0.44, 0.75]; California, Florida, Massachusetts, Michigan, New York, Pennsylvania: U [0.45, 0.75] | n/a |
| Space-reprobing | Transmission rate | β | Alpha (2021-02-14 to 2021-06-19): Wyoming, U [0.54, 0.9]; Iowa, Texas, Washington, U [0.57, 1.12]; California, Florida, Massachusetts, Michigan, New York, Pennsylvania, U [0.585, 1.125];  Delta (2021-06-06 to 2021-12-18): Wyoming, U [0.5, 1.02]; Iowa, Texas, U [0.52, 1.27]; Washington, U [0.53, 1.27]; California, Florida, Michigan, New York, Pennsylvania, U [0.54, 1.275];  Delta_holiday (2021-07-04 to 2021-07-31): Massachusetts, U [0.855, 1.275];  Delta1 (2021-06-13 to 2021-07-03): Massachusetts, U [0.54, 1.275];  Delta2 (2021-08-01 to 2021-08-21): Massachusetts, U [0.54, 1.02];  Delta3 (2021-08-22 to 2021-12-18): Massachusetts, U [0.54, 1.275];  Omicron_BA.1 (2021-12-05 to 2022-03-26): Wyoming, U [0.62, 1.2]; Texas, U [0.65, 1.5]; Iowa, Washington, U [0.66, 1.5]; California, Florida, Massachusetts, Michigan, New York, Pennsylvania, U [0.675, 1.5];  Omicron_BA.2 (2022-03-06 to 2022-05-07): Wyoming, U [0.75, 1.68]; Iowa, Texas, Washington, U [0.79, 2.1]; California, Florida, Massachusetts, Michigan, New York, Pennsylvania, U [0.81, 2.1];  Omicron_BA.2.12.1 (2022-04-03 to 2022-06-18): Wyoming, U [0.75, 1.68]; Iowa, Texas, Washington, U [0.79, 2.1]; California, Florida, Massachusetts, Michigan, New York, Pennsylvania, U [0.81, 2.1];  Omicron_nonBA.1o2 (2022-06-05 to 2022-12-31): Wyoming, U [0.75, 1.68]; Iowa, Texas, Washington, U [0.79, 2.1]; California, Florida, Massachusetts, Michigan, New York, Pennsylvania, U [0.81, 2.1];  wave1 (2020-01-01 to 2020-09-30): Wyoming, U [0.41, 0.6]; Iowa, Texas, Washington, U [0.44, 0.75]; California, Florida, Massachusetts, Michigan, New York, Pennsylvania, U [0.45, 0.75];  wave2 (2020-10-01 to 2021-03-20): Wyoming, U [0.41, 0.66]; Iowa, Texas, Washington, U [0.44, 0.83]; California, Florida, Massachusetts, Michigan, New York, Pennsylvania, U [0.45, 0.825] | n/a |
| Space-reprobing | Infection-detection rate | *r* | 2020summer (2020-06-01 to 2020-10-03): Massachusetts, New York, Wyoming, U [0.05, 0.25]; Michigan, Pennsylvania, U [0.05, 0.3125]; California, Florida, Iowa, Texas, Washington, U [0.05, 0.375];  2021summer (2021-06-01 to 2021-06-30): California, Iowa, Massachusetts, Michigan, New York, Pennsylvania, U [0.02, 0.1]; California, Florida, Iowa, Massachusetts, Michigan, New York, Pennsylvania, Texas, Washington, U [0.03, 0.25];  Alpha (2021-02-14 to 2021-05-31): Florida, Iowa, Massachusetts, Michigan, Pennsylvania, Texas, U [0.2, 0.4]; California, New York, Washington, Wyoming, U [0.2, 0.48];  Delta (2021-05-31 to 2022-01-01): Florida, Texas, Washington, Wyoming, U [0.1, 0.5];  Delta1 (2021-06-21 to 2021-07-17): California, Iowa, Massachusetts, Michigan, New York, Pennsylvania, U [0.1, 0.5];  Delta2 (2021-06-27 to 2021-08-08): California, Iowa, Massachusetts, Michigan, New York, Pennsylvania, U [0.3, 0.5];  Delta3 (2021-07-19 to 2022-01-08): California, Iowa, Massachusetts, Michigan, New York, Pennsylvania, U [0.1, 0.5];  massvax (2021-05-14 to 2021-05-31): California, Iowa, Massachusetts, Michigan, New York, Pennsylvania, Washington, U [0.05, 0.3];  Omicron0 (2021-10-15 to 2021-12-11): all locations, U [0.001, 0.05];  Omicron1a (2021-12-05 to 2021-12-18): all locations, U [0.1, 0.5];  Omicron1b (2021-12-05 to 2021-12-25): all locations, U [0.1, 0.6];  Omicron2 (2021-12-26 to 2022-06-18): all locations, U [0.05, 0.2];  Omicron3 (2022-06-05 to 2022-12-31): all locations, U [0.02, 0.18];  wave1 (2020-03-08 to 2020-05-31): all locations, U [0.08, 0.35];  wave2 (2020-09-06 to 2021-03-20): Florida, Iowa, Massachusetts, Michigan, Pennsylvania, Texas, U [0.12, 0.4]; New York, Washington, Wyoming, U [0.144, 0.48]; California, U [0.144, 0.54] | n/a |
| Space-reprobing | Infection-fatality risk | IFR | 2020summer (2020-06-01 to 2020-09-30): all locations, U [1e-04, 0.005];  Alpha (2021-03-07 to 2021-06-19): all locations, U [1e-04, 0.015];  Delta (2021-06-13 to 2022-01-01): all locations, U [1e-04, 0.015];  massvax (2021-05-14 to 2021-06-26): California, Florida, Iowa, Massachusetts, Michigan, New York, Pennsylvania, Texas, Washington, U [1e-04, 0.01];  Omicron_BA.1 (2021-12-12 to 2022-06-18): all locations, U [4e-05, 0.004];  Omicron_nonBA.1 (2022-06-05 to 2022-12-31): all locations, U [8e-06, 0.0032];  wave1early (2020-03-16 to 2020-04-15): all locations, U [0.005, 0.025];  wave1late (2020-04-16 to 2020-05-31): all locations, U [0.001, 0.015];  wave2 (2020-10-01 to 2021-04-24): all locations, U [2e-04, 0.0125] | n/a |
