# Supplementary figures and images for: Development of Accurate Long-lead COVID-19 Forecast
Source: PLoS Comput Biol. 2023 Jul 17;19(7):e1011278. doi: 10.1371/journal.pcbi.1011278 (PMC10374152; doi:10.1371/journal.pcbi.1011278)

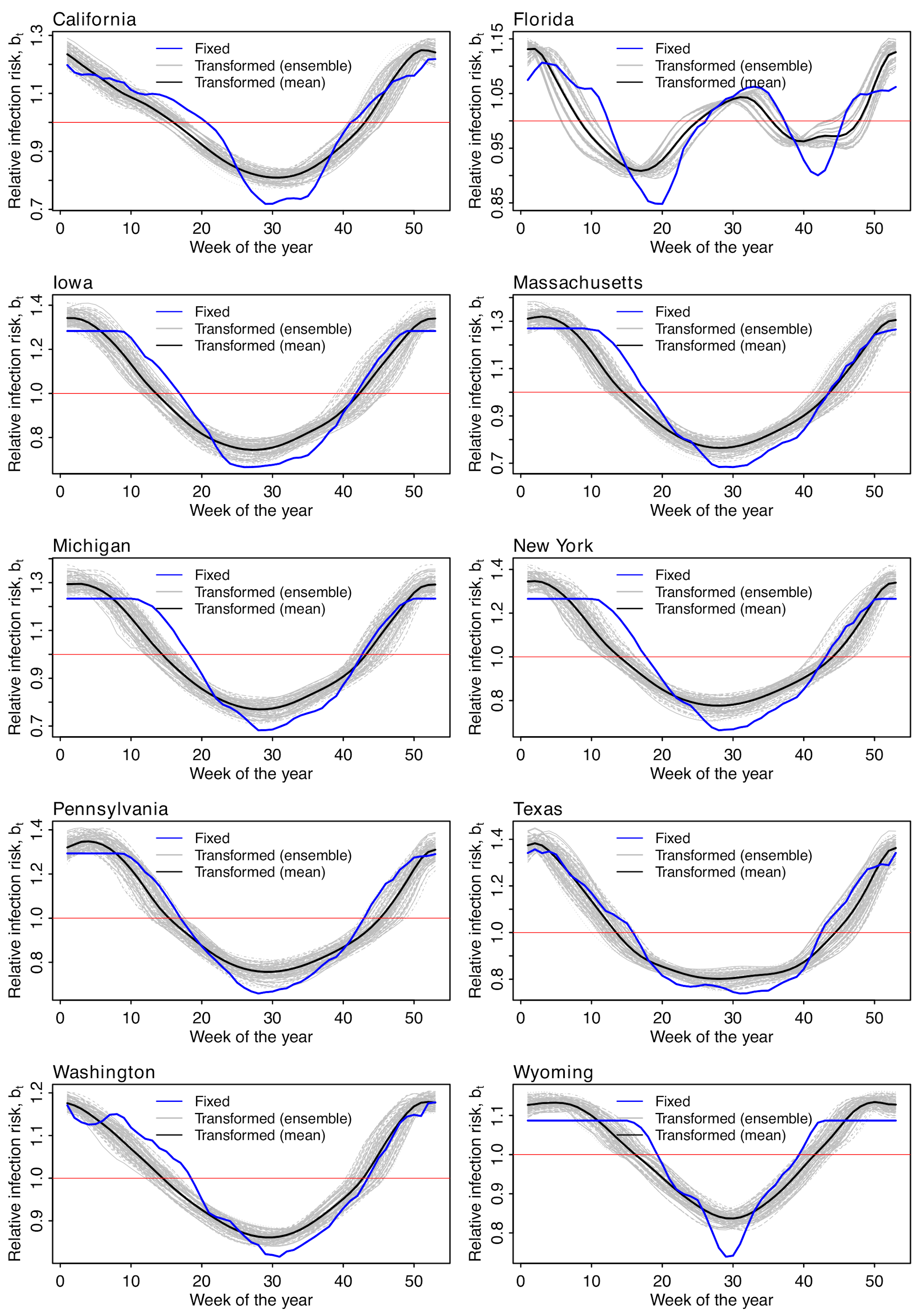

Supplement: S1 Fig — For each state (each panel), the blue line shows the estimated trend of seasonal infection risk using Eqs 3a-b and location weather data (temperature and humidity). Grey lines show 100 examples of the transformed seasonal trends per Eqs 4a-d with parameters randomly sampled from the best parameter ranges (S4 Fig); the black line shows the mean of the 100 example trends. (TIF) [file pcbi.1011278.s002.tif]

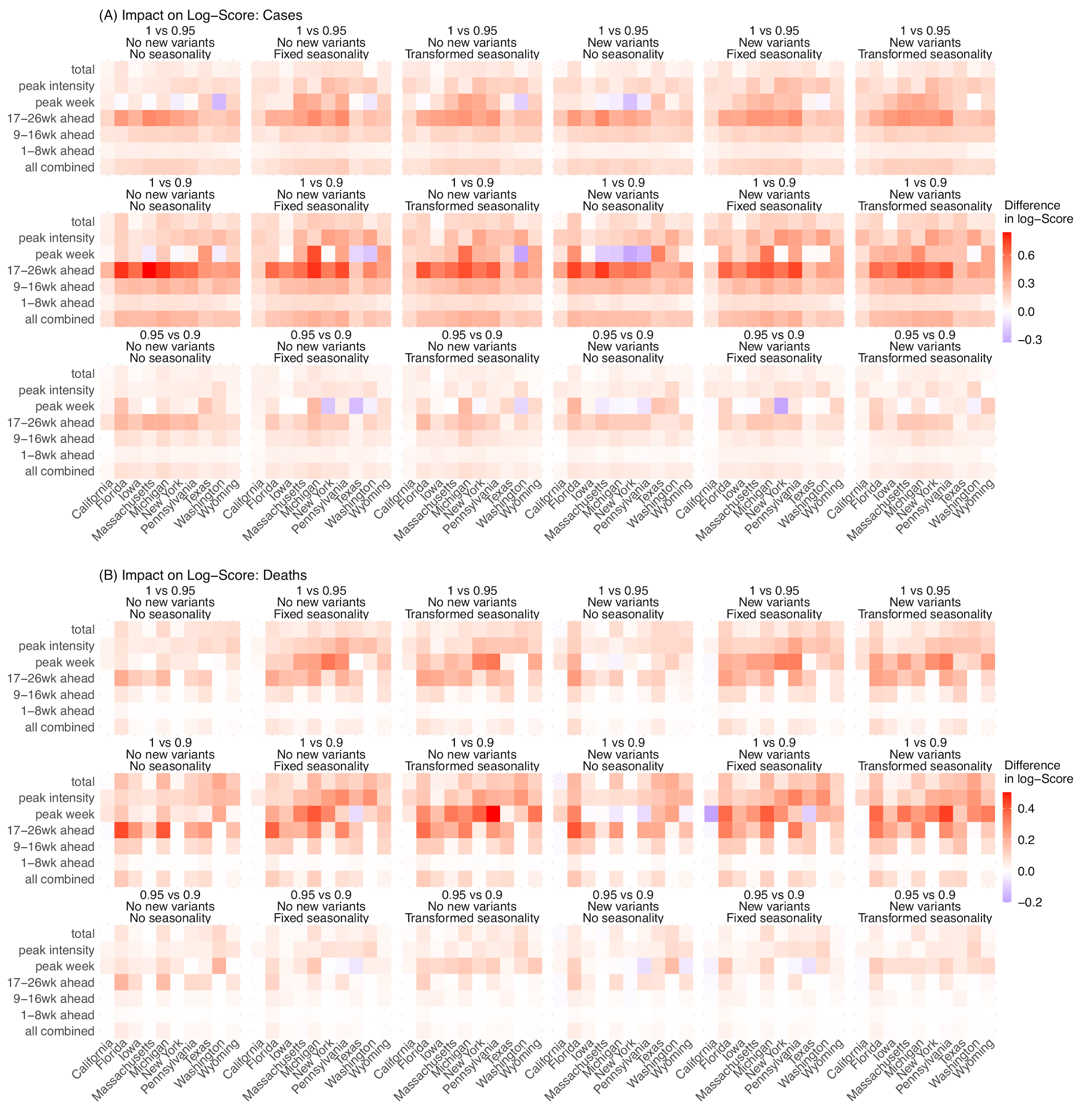

Supplement: S2 Fig — Heatmaps show differences in mean log score for cases (A) and deaths (B), between each forecast approach with different deflation settings (deflation factor γ = 0.95 vs none in the 1st row, 0.9 vs none in the 2nd row, and 0.9 vs 0.95 in the 3rd row; see panel subtitles). Results are aggregated over all forecast weeks for each type of target (y-axis), forecast approach (see specific settings of new variants and seasonality in subtitles), and location (x-axis). For each pairwise comparison (e.g., 0.95 vs none), a positive difference indicates the former approach (e.g., 0.95) outperforms the latter (e.g., none). (TIF) [file pcbi.1011278.s003.tif]

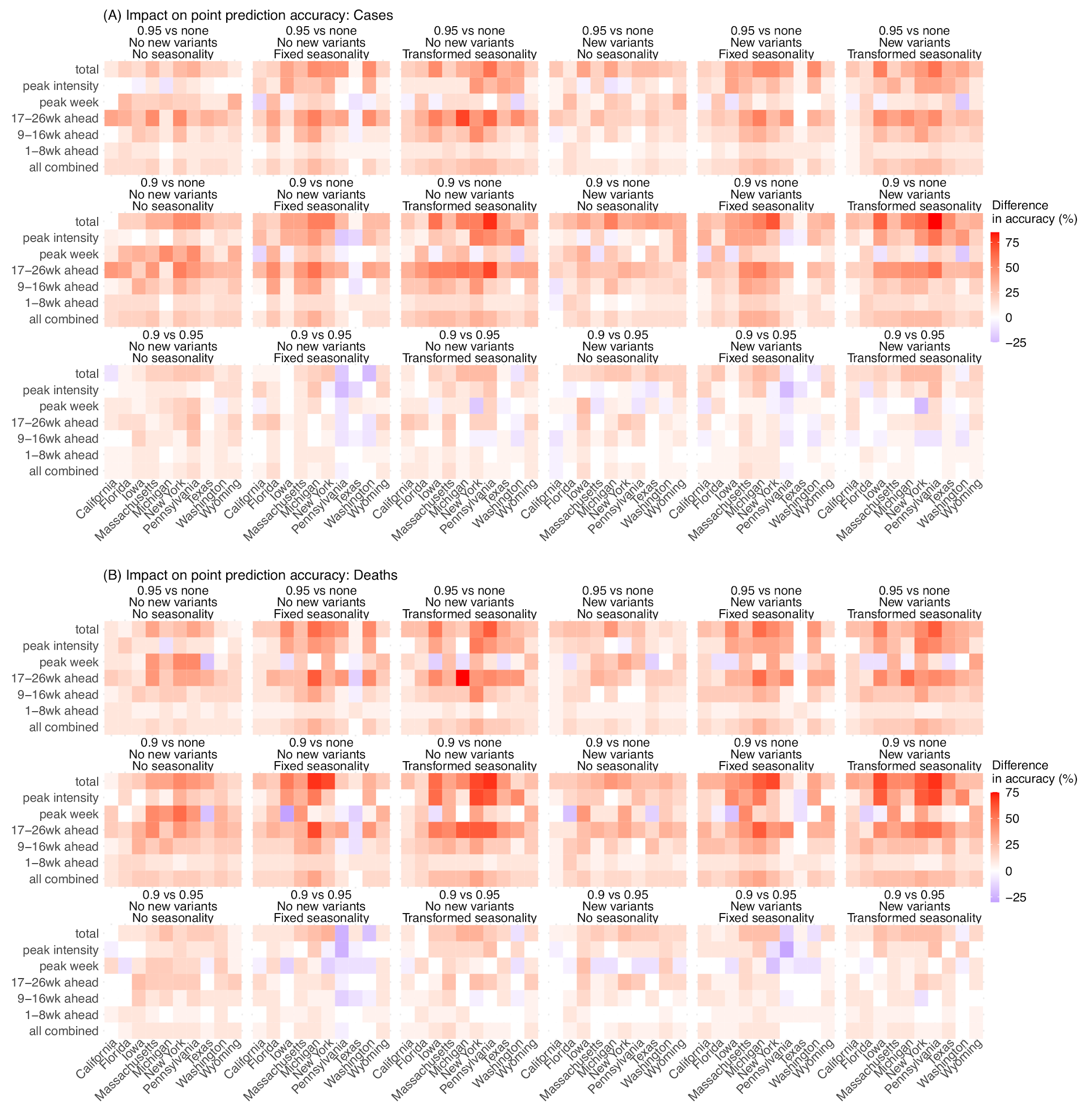

Supplement: S3 Fig — Heatmaps show differences in forecast accuracy of point estimates for cases (A) and deaths (B), between each forecast approach with different deflation settings (deflation factor γ = 0.95 vs none in the 1st row, 0.9 vs none in the 2nd row, and 0.9 vs 0.95 in the 3rd row; see panel subtitles). Results are aggregated over all forecast weeks for each type of target (y-axis), forecast approach (see specific settings of new variants and seasonality in subtitles), and location (x-axis). For each pairwise comparison (e.g., 0.95 vs none), a positive difference indicates the former approach (e.g., 0.95) outperforms the latter (e.g., none). (TIF) [file pcbi.1011278.s004.tif]

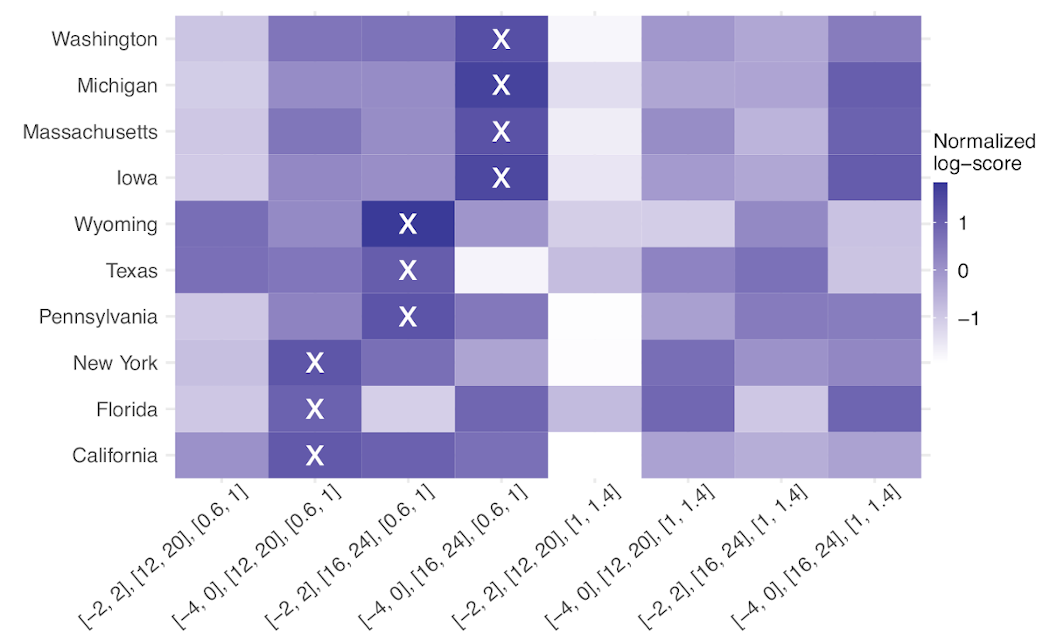

Supplement: S4 Fig — The parameter ranges are shown in x-axis labels for the three parameters in Eq 4a-d (from bottom to top: pshift, δ, and bt, lwr). ‘x’s indicate the best parameter ranges for the corresponding state. (TIF) [file pcbi.1011278.s005.tif]
