# Supplementary material for: Development of Accurate Long-lead COVID-19 Forecast
Source: PLoS Comput Biol. 2023 Jul 17;19(7):e1011278. doi: 10.1371/journal.pcbi.1011278 (PMC10374152; doi:10.1371/journal.pcbi.1011278)
Supplement: Attachment — Submitted filename: reviewer_reply_v2.pdf [file pcbi.1011278.s006.pdf]

MS# PCOMPBIOL-D-22-01834 (Revision)
Title: Development of Accurate Long-lead COVID-19 Forecast

We thank the reviewers for their constructive critiques and suggestions and have revised the manuscript accordingly (changes shown in blue) and provided point-by-point responses to each comment below.

Reviewer #1: In this paper, the authors aim to develop strategies to enhance forecasts of the trajectory of the COVID-19 pandemic. Specifically, an "optimized approach" is used to derive improved predictions 6 months ahead using data for 10 representative US states. My specific comments follow.

1. After reading the paper, I was unclear about the "optimization" framework employed to generate the prediction enhancements. For instance, authors explore three "deflation" factors (1.0, 0.95, 0.9). However, these values were chosen arbitrarily but resulted in positive improvements in forecasting performance relative to their baseline model. If an optimization framework is employed, the model should be calibrated with a training dataset based on the first part of the pandemic (e.g., 2020 and 2021) and then assess forecasting performance based on the most recent pandemic data (2022).

- In this study, the models are optimized/calibrated using the Ensemble Kalman Adjustment Filter (EAKF, Anderson 2001), for all approaches including the baseline approach. That is, for all forecasts, the corresponding model is first trained using all available data up to the week of forecast initiation, prior to generating a forecast. Unlike the offline training (optimization) process used in e.g., computer science, engineering, and econometrics, for infectious disease forecasts, it is common to have a rolling training process, where data are continuously incorporated as new data become available. This rolling training/forecast process is employed in our study for all approaches tested. Please see description on model training (i.e., calibration) in Methods, subsection "Model calibration before forecast generation (i.e. inference)" (Page 12) and details on the forecast process in subsection "Retrospective forecast" (Page 14).

We agree with the reviewer that the model can be calibrated with a training dataset (e.g., data in 2020 and 2021) and the forecast performance assessed using more recent pandemic data (e.g. data in 2022). For short-term (e.g. 1-4 week ahead) forecasts, such a division into a training and testing (performance assessment) period can be done, such as with the rolling training/forecast setting noted above. However, it is not feasible for long-lead forecasts spanning 6 months and a pandemic period of <3 years with dramatically changing dynamics. In particular, the dramatically changing dynamics during the ~3 years of the pandemic – due to non-pharmaceutical interventions and behavioral changes, vaccination rollout, and emergence of multiple new variants – would lead to vastly different situations across the training and testing periods. Thus, we instead examined all rolling forecasts together, and stratified to assess the robustness of the forecast accuracy across different pandemic wave (e.g., the earlier ancestral virus wave, vs different VOC waves) and season (respiratory virus season during Nov –

May) vs. non-respiratory virus season during May – Nov). See discussion of these challenges in e.g., Lines 66 – 85 and 274 – 280.

More importantly, as shown in this study, forecast accuracy differs by approach despite the same optimization/calibration algorithm used for all approaches. This is because the approach based on the three strategies proposed here goes beyond traditional model optimization schemes based on data. Rather, the proposed strategies further address challenges facing long-lead COVID-19 forecasts, i.e., error growth (by deflation), emergence of new variants (by anticipating the impact of new variants), and seasonality (by incorporating long-term seasonal trends). As the best-performing approach is identified by comparing 12 approaches, we had loosely referred to it as the "optimized" approach; however, for the revised manuscript, we now refer to it as the "best-performing approach".

2. Regarding the forecasting horizon, epidemic models have struggled to generate reasonably accurate short-term (up to 4 weeks ahead) predictions of the COVID-19 pandemic. Some real-time and retrospective efforts on this are now well documented as the authors are aware (CDC Forecasting hub), and their forecasting results are publicly available. Hence, it is essential to compare the forecasting performance of any new models/approaches, such as the one presented here, with the historical performance of prior modeling efforts. This is to say that before assessing such long-lead predictions, it would be important to evaluate short-term predictions (e.g., 4-week) and compare the results with those obtained from prior forecasting efforts to gauge the extent of the forecasting performance improvements reported in this paper. Without comparisons with a benchmark model is challenging to assess the importance of the advancement reported using new models or approaches.

- We agree with the reviewer that a comparison with other forecast methods would provide a better assessment of the proposed approach in general. However, as noted in the manuscript, long-lead forecasts – the focus of this study – have not been performed by other groups. While the short-term (1-4 week ahead) forecasts are available, we do not see a substantial difference in forecast performance among our approaches; as such, we do not expect to see substantial differences comparing to other groups' 1- to 4-week ahead forecasts. In addition, to our knowledge, the publicly available 1- to 4-week ahead forecasts from the CDC Forecasting hub are generated in real time whereas the forecasts generated in this study are mostly retrospective with additional information (e.g. mobility) as noted in the manuscript. As such, we do not believe it fair to compare our retrospective forecasts with the CDC co-led real-time forecasts.

Per the reviewer suggestion, we have generated retrospective forecasts using ARIMA models, to provide a more "objective" comparison to the approaches proposed in this study. Please see details in the reply to Comment #3 below.

3. It'd be helpful to to compare their forecasting performance results with a benchmark model such as ARIMA. To what extent the model improves performance relative to more straightforward models? It will help determine how successful the approach is comparable to

other models, even if retrospective, which is critical to advancing the field of epidemic forecasting.

- Per reviewer suggestion, we have generated retrospective forecasts using ARIMA models. We first tested 5 versions of ARIMA(X) model: 1) ARIMA model using either case or mortality data alone, optimized using all data up to the week of forecast initiation, as done for all other approaches; 2) ARIMAX model using case/mortality data and mobility data including for the forecast period (i.e., X = mobility; referred to as "ARIMAX.MOB"); 3) ARIMAX model using case/mortality data and the estimated seasonal trend from the fixed seasonal model in this study (i.e., X = seasonality; referred to as "ARIMAX.SN"); 4) ARIMAX model using case/mortality data, mobility data, and the estimated seasonal trend (i.e., X = mobility and seasonality; referred to as "ARIMAX.MS"); and 5) ARIMAX model using case/mortality data, mobility data, the estimated seasonal trend, and vaccination data (i.e., X = mobility, seasonality, and vaccination; referred to as "ARIMAX.FULL"). Across the entire study period, the ARIMAX.SN (including seasonality) performed the best and is used as a benchmark model for comparison. We have added these results in the revision (see Table S5 in the revision)

Compared to the best-performing ARIMAX.SN model, our baseline approach (i.e., no deflation, no new variants, and no seasonality) performed similarly well whereas our best-performing approach (i.e., applying deflation with γ = 0.9, the new variant rules, and the transformed seasonality form) had much superior performance (Table S6). We have now added these results in the revision (see subsection "Forecast performance compared with ARIMAX models" in Page 9) and Table S6 (included below for reference).

Table S6 (in the revision). Comparison of forecast performance of the approaches developed in this study with the best-performing ARIMAX model. Numbers show the mean log score or point prediction accuracy of forecasts (specified in the "metric" column), aggregated across the entire study period and all locations, for all forecast targets combined or individual forecast targets (specified in the "target" column). Bolded fonts indicate best performance (highest log score or accuracy).

		Log score			Accuracy		
target	measure	ARIMAX.SN	Baseline	Best-Performing	ARIMAX.SN	Baseline	Best-Performing
all	Cases	-2.75	-1.95	**-1.46**	18%	11%	**26%**
all	Deaths	-1.64	-0.97	**-0.65**	21%	17%	**31%**
1-8wk ahead	Cases	-2.04	-1.08	**-0.91**	26%	26%	**38%**
1-8wk ahead	Deaths	-1.27	-0.42	**-0.35**	28%	39%	**48%**
9-16wk ahead	Cases	-2.8	-1.86	**-1.49**	14%	4%	**20%**
9-16wk ahead	Deaths	-1.64	-0.83	**-0.64**	18%	7%	**25%**
17-26wk ahead	Cases	-3.26	-2.8	**-1.87**	12%	1%	**16%**
17-26wk ahead	Deaths	-1.72	-1.43	**-0.8**	**16%**	1%	**16%**

peak intensity	Cases	-4.39	-2.43	**-2.01**	19%	20%	**40%**
peak intensity	Deaths	-3.18	-1.69	**-1.36**	23%	30%	**51%**
peak week	Cases	-3.01	-3.51	**-2.73**	20%	24%	**42%**
peak week	Deaths	-2.36	-2.61	**-1.53**	23%	35%	**56%**
total	Cases	-2.54	-1.05	**-0.75**	13%	7%	**33%**
total	Deaths	-2.29	-0.99	**-0.67**	17%	9%	**36%**

4. A log score is used as the primary metric to assess forecasting performance. However, the gold standard to determine forecasting performance is the weighted interval score, which is a proper score and has been used to evaluate performance in prior forecasting efforts during the COVID-19 pandemic.

- In this study, we used log score and point prediction accuracy (based on the relative error) to assess forecast performance, and both metrics (and more so for log score) have been commonly used to evaluate performance including in many CDC (co-)led efforts (see, e.g., Holcomb et al. 2023 *Parasites Vectors* 16:11; Reich et al. 2019 *PNAS* 116:3146-54; Biggerstaff et al. 2018 *Epidemics* 24:26-33). While binning issues can corrupt the log score, this is not an issue with the uniform application we apply. More generally, multiple scoring methods including log score are widely used to evaluate forecasts, rather than having a certain "gold standard". See a full discussion in Wilks DS. 2011 (*Statistical Methods in the Atmospheric Sciences*. p. 301-94) and Gneiting T & Raftery AE. 2007 (*JASA*. 2007;102:359-78). For example, numerical weather prediction, the exemplar forecast science, has used various skill scores for forecast evaluation; see, e.g., the European Centre for Medium-Range Weather Forecasts (https://charts.ecmwf.int/?facets=%7B%22Type%22%3A%5B%22Verification%22%5D%2C%22Range%22%3A%5B%5D%7D). These scoring methods all serve to evaluate forecast performance. Similarly, for the purpose of comparing forecast approaches examined in this study, we believe the use of log score and point prediction accuracy is appropriate here.

5. Reporting the performance metrics in a supp file will help other teams use your model as a benchmark for comparison purposes.

- We have now posted all data and model code, forecasts done in this study, and the performance metrics on Github (see https://github.com/wan-yang/covid_long_lead_forecast).

6. The description of the epidemic model could be enhanced by providing a table that indicates which parameters are estimated and which are fixed from external information.

- All model parameters estimated for the epidemic model were listed in Table S5 in the original manuscript and now Table S8 in the revision.

7. The paper's title should indicate that the forecasts are based on a retrospective analysis.

- This paper focuses on the development of accurate long-lead forecast method. While the forecast performance is evaluated primarily based on retrospective forecasts (as clearly noted in the abstract and the manuscript throughout), the approach developed here applies to real-time forecasts, and some of forecasts generated in this study are real-time forecasts. In particular, per Reviewer 2's suggestion, we have now added results of the last forecasts we generated for this study, which were done in real time at the time of forecast initiation (see new subsection "Forecast for the 2022 – 2023 respiratory virus season" in Page 9, new figures 8-9 and Table S7 in the revision). Therefore, we believe the title of the paper ("Development of accurate long-lead forecast") accurately describes the study.

Reviewer #2: The authors present a very nice and comprehensive study exploring model improvements to build a better COVID-19 forecasting model. The paper is very well written and highly detailed, yet interesting and readable. While I do not expect any major revisions, there are a couple concerns that I would like to see addressed.

First, the authors made the choice to use data available and even fitted separately from the "future" forecast period. While I understand the choice to do this in their analysis aimed to understand how each of the 3 components they were evaluating contributed to forecast accuracy, I would like to see either a little more text discussing this, or even better, a sensitivity analysis where those future data are not used, and instead they are either predicted or used from the point of forecast. While it may not make a major difference, these predictions may interact with the impact of each of these components. Further, for readers who may not read in depth, they may misinterpret the model as being much more accurate than it would be to forecast a long horizon. This should be made more explicit.

- We agree with the reviewer that it should be made clear that these forecasts are done retrospectively and have emphasized this repeatedly throughout the paper (abstract, text, and figure/table captions). In this study, we focus on comparing the approaches based on the *relative* improvement of either log score or accuracy (e.g. Figures 3 – 6 and Table 1). Whenever we present specific forecast accuracies, we clearly state that the numbers are measured based on retrospective forecasts in the text (see e.g., Lines 313 – 316 in the Results, see excerpt below) and all related figure/table captions (i.e., Fig 7 and Table 2). In addition, we specifically discuss the retrospective nature of these forecasts (see Lines 401 – 407 and excerpt below):

"… We note the forecasts here were generated retrospectively with information that may not be available in real time and thus likely are more accurate as a result." (Lines 313 – 316)

"To focus on the above three challenges, <u>in our retrospective forecasts</u>, we used data/estimates to account for several other factors shaping COVID-19 dynamics. These included behavioral changes (including those due to NPIs), vaccination uptake, changing detection rates and hence case ascertainment rate, as well as changes in infection fatality risk due to improvement of treatment, vaccination, prior infection, and differences in the innate virulence of circulating variants. <u>For real-time forecast, such data and estimates would likely not be available and thus forecast accuracy would likely be degraded."</u> (Lines 401 – 407)

Further, for this revision, 6 months has passed since we generated the forecasts and we are now able to evaluate the last forecasts done in real time at the time of the initial study. We have added a subsection to present the real-time forecasts and a preliminary assessment. See Lines 327 - 342 and excerpt below:

"Forecast for the 2022 – 2023 respiratory virus season

Figs 8-9 present real-time forecasts of October 2022 – March 2023 for the 10 states, and Table S7 shows a preliminary accuracy assessment based on data obtained through March 31, 2023. Accounting for under-detection, large numbers of infections (i.e., including undocumented asymptomatic or mild infections) were predicted in the coming months for most states; predicted attack rates over the 6-month prediction period ranged from 16% (IQR: 7 – 31%) in Florida to 30% (IQR: 15 – 47%) in Massachusetts (Fig 9). Relatively low case numbers and fewer deaths at levels similar to or lower than previous waves were forecast, assuming case ascertainment rates and infection-fatality risks similar to preceding weeks (Fig 9). Compared to data reported 6 months later (i.e., not used in the forecasts), the weekly forecasts in general captured trajectories of reported weekly cases over the 6 months for all 10 states (Fig 8, middle column for each state) but under-predicted deaths for half of the states (i.e., New York, Massachusetts, Michigan, Wyoming, and Florida; Fig 8, right column for each state). For the cumulative totals, predicted IQRs covered reported tallies in all 10 states for cases and the majority of states for deaths, while the 95% predicted intervals covered reported cumulative cases and deaths in all states (Fig 9)."

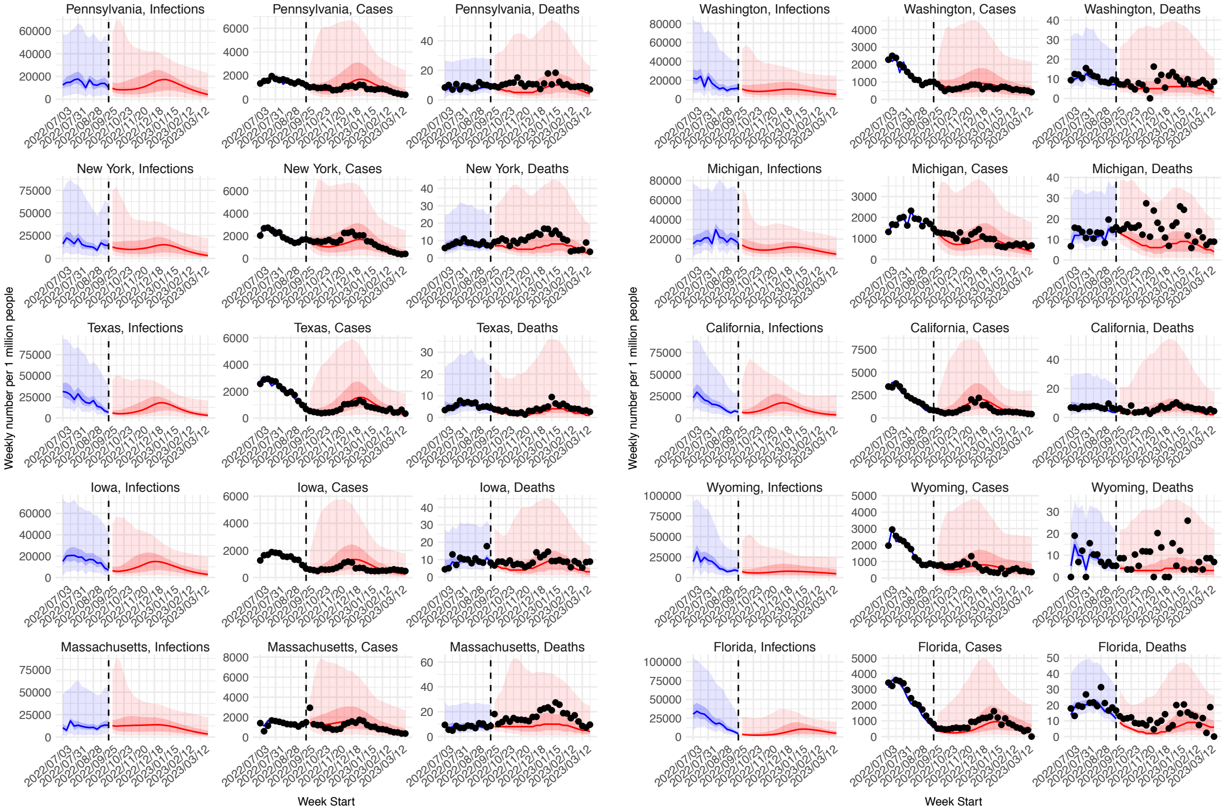

Fig 8 (in the revision). Real-time forecasts for the 2022-2023 respiratory virus season. The states are arranged based on accuracy of historical forecast (higher accuracy for those in the left panel and those on the top). In each panel, each row shows estimates and forecasts of weekly numbers of infections (1st column), cases (2nd column), or deaths (3rd column) for each state. Vertical dashed lines indicate the week of forecast initiation (i.e., October 2, 2022). Dots show reported weekly cases or deaths, including for the forecast period. Blue lines and blue areas (line = median; darker blue = 50% CI; lighter blue = 95% CI) show model training estimates. Red lines and red areas (line = median; dark red = 50% Predictive Interval; lighter red = 95% Predictive Interval) show model forecasts using the best-performing approach.

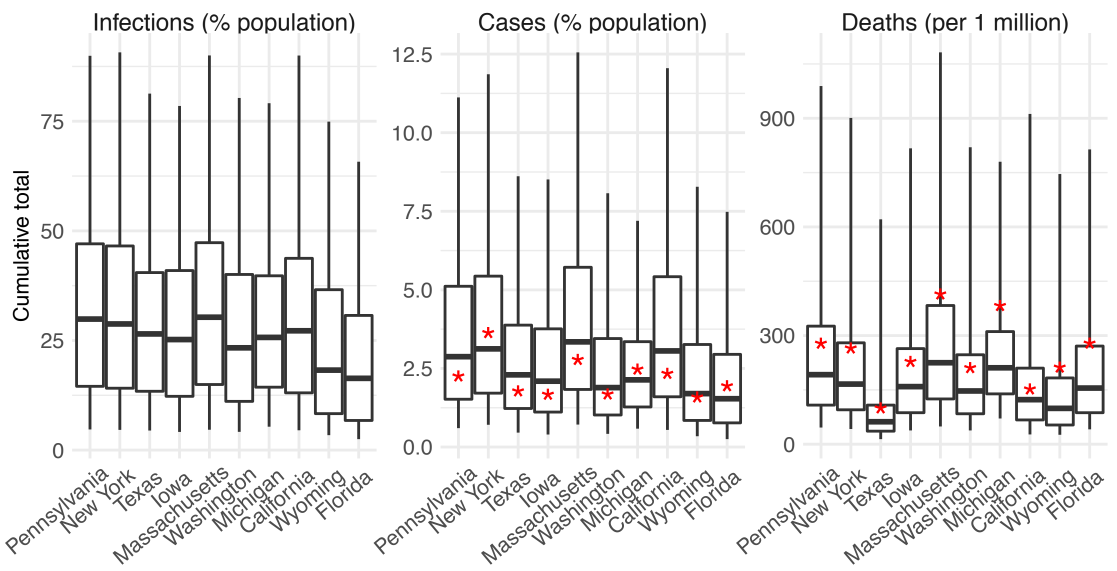

Fig 9 (in the revision). Real-time forecasts of cumulative infections, cases, and deaths during the 2022-2023 respiratory virus season. Box plots show distributions of predicted total number of infections (1st panel, scaled to population size; i.e. attack rate), cases (2nd panel, scaled to population size), and deaths (3rd panel, scaled per 1 million persons) from the week starting 10/2/2022 to the week starting 3/26/2023. Thick line = median; box edge = interquartile range; whisker = 95% prediction interval. The states (x-axis label) are arranged according to accuracy of historical forecast (higher accuracy from left to right). Red asterisks (*) show reported cumulative cases and deaths during the forecast period.

Second, it is not clear to me why specific periods of the pandemic are not included in the analysis, in particular from August - November 2021. While this period may have been challenging due to the rise in Omicron, it seems a little unfair to not present a period because the model did not perform well during it, if that is the case. Either a reason for exclusion should be made clear, or it should be included.

- Please note the August – November 2021 period is included in the evaluation. For the pre-Omicron period, the last forecasts were *initiated* in mid-August, 2021, which covered the period of August 2021 – February 2022 because each forecast spans 6 months. This is explained in the Supplemental text (see Lines 187 – 198). To clarify, we have also added a brief note in the revised main text (see Lines 508 – 510 and excerpt below):

"For all states, we generated retrospective forecasts of weekly cases and deaths 26 weeks (i.e., 6 months) into the future for the non-Omicron period and the Omicron period, separately. For the non-Omicron period, we initiated forecasts each week from the week of July 5, 2020 (i.e., after the initial wave) through the week of August 15, 2021. Note that because each forecast spans 6 months, the last forecasts initiated in mid-August 2021 extend to mid-Feb 2022, covering the entire Delta wave (see Supplemental text for details)…"
