# Supplementary material for: Development of Accurate Long-lead COVID-19 Forecast
Source: PLoS Comput Biol. 2023 Jul 17;19(7):e1011278. doi: 10.1371/journal.pcbi.1011278 (PMC10374152; doi:10.1371/journal.pcbi.1011278)
Supplement: Attachment — Submitted filename: Reviewer_reply_r2.docx [file pcbi.1011278.s007.docx]

MS# PCOMPBIOL-D-22-01834R1 (Revision)

Title: Development of Accurate Long-lead COVID-19 Forecast

We thank the reviewer for the comments and have revised the manuscript accordingly (changes highlighted in blue) and provided point-by-point responses to each comment below.

Reviewer #1: Thanks for the clarifications, the extension of the study to incorporate real-time forecasts, and the additional benchmark comparison with the ARIMA forecasts, which I believe is an essential addition to the paper indicating that the method presented outperformed ARIMA forecasts.
I only have a few residual comments for the authors’ consideration.

1) While the scope of the authors’ work is on long lead forecasts, I did not entirely agree that it would be unfair to retrospectively compare the performance of new models/approaches with the results of prior real-time studies, such as the CDC COVID-19 forecasting hub so long the new methods are fully documented/reproducible. The findings should also be presented as retrospective rather than derived in real-time.

- We agree that, if retrospective forecasts are done in a way mimicking real-time forecasts (in particular, using data released in real time without revision and only data prior to forecast initiation), then such retrospective forecasts can be compared to real-time forecasts. However, as described in the manuscript (see Methods, Lines 516 – 529, and except below), here we also used additional model estimates and mobility and vaccination data including those for the forecast period, which would not be available in real time. This choice is to reduce uncertainty from the rapid changes in infection detection rate, infection-fatality risk, and public health interventions (in particular, social distancing and vaccination) during the pandemic, that could obscure the underlying infection rate and forecast accuracy. Because these estimates and data were used in all approaches tested in our study, it is a fair comparison across the study approaches, and would allow us to focus on comparing the different approaches. However, as we stated previously, it will be unfair to compare the retrospective forecasts generated here to real-time forecasts done without such additional information. We do not wish to promote such unfair comparison, especially when our results would have an unfair advantage over others.

“To generate a forecast, we ran the model-inference system until the week of forecast, halted the inference, and used the population susceptibility and transmissibility of the circulating variant estimated at that time to predict cases and deaths for the following 26 weeks (i.e., 6 months). Because the infection detection rate and infection-fatality risk are linked to observations of cases and deaths (see the SI), changes of these quantities during the forecast period could obscure the underlying infection rate and forecast accuracy. Thus, for these two parameters specifically, we used available model-inference estimates for corresponding forecast period weeks to allow comparison of model-forecast cases and deaths with the data while focusing on testing the accuracy of different model settings (e.g., seasonality and new variant settings). For the same reason, we used all available mobility and vaccination data including those for the forecast period, which would not be available in real time. For weeks in the future without data/estimates, we used the latest estimates instead… “ (Lines 516 – 529)

We present all retrospective forecasts as retrospective throughout the manuscript (in total, 19 instances referring to the relevant forecasts as retrospective; see highlighted text). Per the reviewer suggestion, we have also revised the Author Summary to make it clear that the testing is done based on retrospective forecasts:

“Comprehensive testing based on retrospective forecasts shows the forecast approach using all three strategies consistently outperforms a baseline approach without these strategies across different variant waves and locations in the United States for all forecast targets, improving the probabilistic forecast accuracy by ~50% and point prediction accuracy by ~100%.” (Lines 37 – 41, Author Summary)

2) Regarding the performance metrics, as noted previously more recent forecasting studies including the CDC COVID-19 forecasting hub have based the performance evaluation on the weighted interval score (WIS) while previous forecasting studies, including those noted by the authors, used to focus performance on the log score as indicated by the authors. The authors may agree that using a standard set of performance metrics would allow authors to systematically compare performance across different methods/approaches that may be developed subsequently.

--Multiple performance metrics are widely used (e.g., in numerical weather forecast and infectious disease forecast), and all serve to evaluate forecast performance. In addition, different metrics may have different advantages over one another. For example, as noted in our manuscript (see Method, Lines 560 – 561), log score, when exponentiated, can be intuitively interpreted as the probability correctly assigned to the bins containing the observations. See a full discussion of the pros and cons of different metrics in Wilks DS. 2011 (*Statistical Methods in the Atmospheric Sciences*. p. 301-94). In the past several decades, numerical weather forecast has become highly accurate, in a large part due to many researchers contributing with diverse views and expertise. In comparison, infectious disease forecast, which has progressed tremendously in the past decade, is still in an early stage. Both authors here have worked extensively in the field of infectious disease forecast and wish to see the field progress as numerical weather forecast did, benefiting from diverse viewpoints and expertise.

For researchers interested in comparing methods including the ones presented here, we have published full model code for reproduction of our methods (https://github.com/wan-yang/covid_long_lead_forecast). Researchers wishing to use other performance metrics (e.g., WIS) can freely modify the model code, tailoring the evaluation metric to their own need.

Lastly, as noted in the manuscript and the reply to comment #1, most forecasts in this study were carried out retrospectively with additional information not available in real time, and thus should not be compared with real-time forecasts.
